# Supplementary material for: Impact of COVID-19 pandemic on breast cancer screening in a large midwestern United States academic medical center
Source: PLoS One. 2024 May 20;19(5):e0303280. doi: 10.1371/journal.pone.0303280 (PMC11104587; doi:10.1371/journal.pone.0303280)
Supplement: S1 File — (DOCX) [file pone.0303280.s001.docx]

**Supplementary methods**

**I. Selection of study population from the EPIC database.**

We initially obtained EPIC data for all valid female patients who showed up for an appointment, lived in Missouri or Illinois, and were placed on a registry that tracks their history for wellness markers (annual visits, mammograms, etc). Next, we excluded patients who had a previous bilateral mastectomy (ICD-10 code Z90.13) and those who we recorded as having undergone a simultaneous bilateral mastectomy.  We also removed patients who had separate right (ICD-10 code Z90.11) or left (ICD-10 code Z90.12) mastectomies. We removed patients who had a medical exclusion listed as the reason they did not get a mammogram; those with health modifiers that listed them as trans women, having double mastectomies, or already having cancer; and women with special-needs care or in a long-term facility. We formulated a list of orders in final status that had a visit type associated with breast cancer screening at Siteman Cancer Center and occurred after the patient’s 21^st^ birthday.  Our final patient list was non-excluded patients who had at least one of these orders. This dataset was the parent dataset used to derive our analytic datasets.

**II. Selection of the study population to create analytic datasets.**

To arrive at our analytic datasets (main Bayesian state-space models analyses and age category and race/ethnicity attrition analyses), we further excluded encounters among women who did not reside in one of 247 zip codes in Missouri and Illinois that comprise the BJC catchment area (see **Supplemental data**), mammogram encounters marked as diagnostic, non-unique encounters based on patient ID and procedure ID, encounters not linked to an identifiable mammogram facility site in the St. Louis Region, those in females who were < 40 years old on the date of their mammogram screening encounter, and those occurring in 2018 for our main analyses. From this dataset, we derived all analytic datasets. For our Bayesian state-space models analyses dataset we further excluded encounters in 2021 and those whose race/ethnicity was not classified as NH White, NH Black or African American, NH Asian or Hispanic due to four or fewer encounters per week in these groups. For our main age and race/ethnicity attrition analyses, we arrived at our analytic datasets by first identifying whether a woman had one or more screening encounters in each year from 2019 to 2022. We next excluded the second record for women who had more than one encounter in a single year (based on identical values for patient ID, year, race/ethnicity category, and median income quartile), women who did not have a screening encounter in 2019 and those with missing zip code median household income yielding our age attrition analysis dataset. For our race/ethnicity attrition analysis, we further excluded those whose race/ethnicity was not classified as NH white, NH Black or African American, NH Asian or Hispanic due to small numbers. Exclusions are detailed step by step to arrive at each analytic dataset in **Figure 1**.

**III. Statistical models**

# **Spectral decomposition**

To model seasonal trends, we take a parsimonious approach as recommended by Cryer and Chan [1] and attribute the ebb and flow of visits throughout the year to the formulation:

$$\mu_{s}=\beta_{A}\cos\left( 2\pi f+\rho\right),$$

where $f$ is the value that describes the number of weeks past the origin point of the study divided by the number of weeks in a year and $\rho$ is a shift term. The above lets us model dips and valleys in screening mammograms as a smooth function instead of including a different term for every month.

# **Descend/ascend weighting terms**

Dummy regressions are excellent for their simplicity, but quite inflexible – for a recent treatment of this issue in the differences in differences literature, see this article [2]. In light of this, we used a simple term for an averaging component that describes how we “descend into” and “ascend out of” the acute pandemic phase. Here, we take advantage of the probit function and specify

$$\tau_{t,k}=1-\Phi\left( \alpha_{k}+(t- t_{d})\cdot\gamma_{k} \right)+\Phi\left( \xi_{k}+(t-t_{a})\cdot\nu_{k} \right),$$

where $t$ is the time, $t_{d}$ and $t_{a}$ are time variables offset such that the center of the descend and ascend periods are set at $(t- t_{d})=0$ and $(t-t_{a})=0$ respectively. The probit offers a wide variety of shapes; we can achieve anything from near-linear increases to an “S-shaped” curve. This gives us a flexible class of weights that appropriately average the effect of the acute pandemic process on the observations in our model.

# **State–space evolution**

We reflect the time-varying nature of mammogram screenings by group by specifying a state-space model to estimate the group means. Let $T$ be the final time period represented in the data. We then let the mean process representing what happens without the acute pandemic effect evolve as

$$\boldsymbol{\beta}_{t}\sim N(\beta_{t-1}, \boldsymbol{\Sigma}_{\beta})$$

for all $t\in(2, \ldots, T)$. For the acute pandemic process, we take a simpler approach to covariance estimation due to identifiability issues, but a similar random walk approach such that, for the final possible acute pandemic time $P\leq T$, we specify

$$\boldsymbol{\omega}_{t}\sim N\left( \boldsymbol{\omega}_{t-1}, \boldsymbol{D}_{drop} \right)\boldsymbol{,}$$

for all $t\in\left( p+1, P \right)$where $\boldsymbol{D}_{drop}$ is the $k\times k$ matrix with diagonal components $\boldsymbol{\sigma}_{drop}^{2}$ and $0$ in all off diagonals and $p$ is the index for the first week that the pandemic effects are allowed to occur in the model.

# **Prior parameterization**

We initialize the latent $k\times1$state vector $\boldsymbol{\beta}_{1}$ with the prior

$$\boldsymbol{\beta}_{1}\sim N\left( \boldsymbol{2}, 3^{2}\times\boldsymbol{I} \right),$$

Where $\boldsymbol{I}$ is the $k\times k$ identity matrix.

This prior is relatively diffuse given the application. Because we are thinking about mammogram counts, we are stating that our prior belief in week one, we are 95% certain that the average number of screening mammograms for any given group is in the interval (0.02, 2643.87) calculated according to the specified prior of N(2, 3^2) or exp(2 - 3 * 1.96) = 0.02 and exp(2 + 3 * 1.96) = 2643.87. Aside from the weeks where we observe 0 counts, all observations for every group fall into this interval. We set a prior for the drop in visits at

$$\boldsymbol{\omega}_{p}\sim N\left( \boldsymbol{-1}\cdot\boldsymbol{\beta}_{p-1} , \sigma_{drop}^{2} \right),$$

where $p$ is the index for the first week that the pandemic effects are allowed to occur in the model. Holiday and spectral $\beta$ terms are both given a $N(0, 3)$ prior, the spectral shift parameter $\rho$ is given a $Beta\left( 3, 3 \right)$ prior, and all parameters in the section concerning descend/ascend weighting terms are given diffuse $N(0, 10)$ priors. For our state evolution processes, we specify diffuse priors for the overall variance such that

$$\boldsymbol{\sigma}_{drop}^{2}, \boldsymbol{\sigma}_{\beta}^{2}\sim InvGamma\left( 1, 1 \right),$$

and use the LKJ correlation matrix density and an additional parameter describing how regularized our covariance matrix $\boldsymbol{\Sigma}_{\beta}$ is. Here,

$$\eta\sim InvGamma\left( 2, 1 \right).$$

For $\eta\in(0, \infty$), when $\eta=1$, we impose a uniform distribution on all correlation matrices $\boldsymbol{L}_{\beta}\boldsymbol{L}_{\beta}^{T}$, and any $\eta>1$ encourages regularization), and

$$\boldsymbol{L}_{\beta}\sim LKJ\left( \eta\right).$$

We then construct the correlation matrix

$$\boldsymbol{\Sigma}_{\beta}=\boldsymbol{L}_{\beta}\boldsymbol{D}_{\beta}\boldsymbol{L}_{\beta}^{T}$$

where $\boldsymbol{D}_{\beta}$ is the $k\times k$ matrix with diagonal components $\boldsymbol{\sigma}_{\beta}^{2}$ and $0$ in all off diagonals.

**References**

1. Cryer JD, Chan K-S (2008) [CN]Time Series Analysis: With Applications to R. 487. https://doi.org/10.1007/978-0-387-75959-3/COVER

2. de Chaisemartin C, D’Haultfœuille X (2020) Two-Way Fixed Effects Estimators with Heterogeneous Treatment Effects. American Economic Review 110:2964–96. https://doi.org/10.1257/AER.20181169
