# Supplementary material for: Impact of COVID-19 pandemic on breast cancer screening in a large midwestern United States academic medical center
Source: PLoS One. 2024 May 20;19(5):e0303280. doi: 10.1371/journal.pone.0303280 (PMC11104587; doi:10.1371/journal.pone.0303280)
Supplement: S2 File — (RTF) [file pone.0303280.s002.rtf]

---title: "COVID screening paper"author: "Kim Johnson and Caitlin O'Connell"date: output: html_document---# Load packages and open libraries ```{r}pacman::p_load(readr, writexl, dplyr, ggplot2, tidyverse, lubridate, table1, formattable, fable, tsibble, feasts, urca, MASS, lmtest, broom, sandwich, multcomp, openxlsx, eeptools, DiagrammeR, readxl, nnet, odds.n.ends)```# Import files```{r, warning = FALSE}# import encounters dataEncounters <- read_tsv("20178 Johnson COVID Cancer Screening Second Request 20220324 Encounters.txt", show_col_types = FALSE)nrow(Encounters)# import demographics dataDemographics<- read_tsv("20178 Johnson COVID Cancer Screening Second Request 20220324 Demographics.txt", show_col_types = FALSE)# import zip code data from BJC that includes catchment zipcodes used for reportingzipcodes <- read_xlsx("ZIP and MSA Territories.xlsx")```# Data management to create variables needed for analyses```{r}# collapse race categoriesDemographics <- Demographics %>%  mutate(race_col = if_else(ETHNICITY == "Hispanic", 7,                     if_else(RACE=="White", 0,                    if_else(RACE=="Black or African American", 1,                     if_else(RACE=="Asian", 2,                    if_else(RACE=="American Indian or Alaska Native", 3,                    if_else(RACE=="Other Pacific Islander", 4,                    if_else(RACE=="Other", 5,                    if_else(RACE=="Declined"  | RACE=="Unable to Answer" | RACE=="Unknown" | RACE=="Declined~Declined" | RACE=="Declined~Unknown" | RACE=="Unknown~Unable to Answer",NA_real_, if_else(grepl("~",RACE), 6, NA_real_)))))))))) %>%  mutate(race_col = factor(race_col, levels=c(0:7), labels=c("non-Hispanic White", "non-Hispanic Black or African-American",  "non-Hispanic Asian", "Non-Hispanic American Indian or Alaska Native", "non-Hispanic Other Pacific Islander", "non-Hispanic Other", "non-Hispanic Two or more", "Hispanic")))# collapse marital statustable(Demographics$MARITAL_STATUS)Demographics <- Demographics %>%  mutate(marital = case_when(MARITAL_STATUS %in% c("Civil Union", "Married", "Life Partner") ~ 0,                             MARITAL_STATUS %in% c("Divorced", "Legally Separated", "Single", "Widowed") ~ 1,                             MARITAL_STATUS %in% c("Unknown")|is.na(MARITAL_STATUS) ~ 2))# check variabletable(Demographics$marital, Demographics$MARITAL_STATUS, useNA = "always")Demographics$marital <- factor(Demographics$marital, levels = c(0:2), labels = c("Married", "Single", "Unknown/Missing"))```# Identify screening mammograms in Encounters data```{r screening mammograms, include=FALSE}# create new variable to identify screening mammogramsEncounters <- Encounters %>%  mutate(screening = if_else(grepl("screening", PROCEDURE_NAME, ignore.case = TRUE), 1, 0)) %>%  mutate(screening = as.factor(as.character(screening)))# checktable(Encounters$screening)```# Clean variables```{r}# clean date variable to get rid of time stampEncounters <- Encounters %>%  mutate(procedure_date = as.Date(ymd_hms(PROCEDURE_DATE)))# create month variableEncounters <- Encounters %>%  mutate(month = month(procedure_date, label = TRUE))# create year variableEncounters <- Encounters %>%  mutate(year = year(procedure_date))```# Filtering out zipcodes not in MSA territories ```{r, warning = FALSE}zipcodes <- zipcodes %>%  filter(`MSA Sub-Territories` != "Non-MSA") %>% # yields BJC catchment areas    dplyr::select(`Zip Code`) # n = 247```# Exclusions# Filtering out encounters not in zip codes in BJC catchment area```{r}# add new column of the 5-digit zip code in Demographics fileDemographics$zip_5dig <- substr(Demographics$ZIP, 1, 5)# check new variablesummary(as.factor(Demographics$zip_5dig))# add variable indicating the zip is in the included areaDemographics$in_zip <- if_else(Demographics$zip_5dig %in% zipcodes$`Zip Code`, 1, 0)# check new variablesummary(as.factor(Demographics$in_zip))# filter demographics for those in included zip codesDemo_zipcode <- Demographics %>%  filter(in_zip == 1)# filter Encounter dataset for those in zip code catchment Encounters$in_zip <- if_else(Encounters$Epic_MRN %in% Demo_zipcode$Epic_MRN, 1, 0)Encounters_zip <- Encounters  %>%  filter(in_zip == 1) n <- nrow(Encounters_zip)# first exclusion nn_ex1= nrow(Encounters) - nrow(Encounters_zip)n_ex1```# Filtering out encounters not identified as screening mammograms```{r}# create new dataframe with only screening mammogramsscreen_mam_zip <- Encounters_zip %>%  filter(screening == 1)# second exclusion nn_ex2 <- nrow(Encounters_zip) - nrow(screen_mam_zip)n_ex2```# Filtering out duplicate records that have the same MRN and order procedure ID```{r}# this code deletes duplicates that have same MRN and procedure date. screen_mam_zip <- screen_mam_zip %>%  dplyr::select(-ORDER_PROC_ID)screen_zip_unique_a <- unique(screen_mam_zip)# third exclusion nn_ex3 <-  nrow(screen_mam_zip)- nrow(screen_zip_unique_a) n_ex3```# Filtering out sites that cannot be identified as imaging sites```{r}# import crosswalk file from BJCcrosswalk <- read_excel("Breast Imaging Dept Crosswalk_04.05.2023.xlsx") # add variable indicating the department is includedscreen_zip_unique_a <- screen_zip_unique_a %>%  mutate(include_dept = if_else(DEPARTMENT_NAME %in% crosswalk$`Encounter Department Name`,1,0))# check new variablesummary(as.factor(screen_zip_unique_a$include_dept))# filter encounters for those in included departmentsscreen<- screen_zip_unique_a %>%  filter(include_dept == 1)n <- nrow(screen)nsummary(as.factor(screen$DEPARTMENT_NAME)) # 22 departments# fourth exclusion nn_ex4 <- nrow(screen_zip_unique_a)- nrow(screen) n_ex4```# Add in demographics, filter those <40```{r}# create one data set with screening info and demographicsscreen_demo <- left_join(screen, Demographics, by = 'Epic_MRN')# calculate age from birthdatescreen_demo <- screen_demo %>%  mutate(age = floor(age_calc(BIRTH_DATE, procedure_date, units = "years")))# create age at screen categoriesscreen_demo <- screen_demo %>%  mutate(age_cat = case_when(age>=40 & age <50 ~ 0,                             age>=50 & age <60 ~ 1,                             age>=60 & age <70 ~ 2,                             age>=70 & age <80 ~ 3,                             age>=80 ~ 4)) %>%  mutate(age_cat = factor(age_cat, levels=c(0:4),                           labels=c("40-49", "50-59", "60-69", "70-79", "≥0")))# filter out those under 40 years oldscreen_demo <- screen_demo %>%  filter(age >= 40)n <- nrow(screen_demo)n# fifth exclusion nn_ex5 <- nrow(screen) - nrow(screen_demo)n_ex5```# Add in social explorer data and create zipcode quartiles for zipcodes in set# Import data- generated from : Social Explorer Tables: ACS 2021 (5-Year Estimates)(SE), ACS 2021 (5-Year Estimates), Social Explorer; U.S. Census Bureau at https://www.socialexplorer.com/tables/ACS2021_5yr/R13442731on 9/8/2023```{r}R13436768_SL860 <- read_delim("R13442731_SL860_limited.csv",  show_col_types = FALSE)```# Clean up file ```{r}# delete first row that is an extra header rowACSdata <- R13436768_SL860[-1,]```# Reduce ACSdata file to included zipcodes```{r, warning=FALSE}ACSdata <- ACSdata %>%filter(`5-digit ZIP Code Tabulation Area` %in% screen_demo$zip_5dig) # 201 zip codes present # rename median household incomeACSdata <- ACSdata %>%  rename(MedianIncome2021 = `Median Household Income (In 2021 Inflation Adjusted Dollars):`)class(ACSdata$MedianIncome2021)```# Categorize median income into quartiles```{r}# first define binary median income variableACSdata$MedianIncome2021 <- as.numeric(ACSdata$MedianIncome2021)summary(ACSdata$MedianIncome2021)ACSdata <- ACSdata %>%  mutate(median_income_quartile = case_when(MedianIncome2021 > 84953 ~ 3,                                          MedianIncome2021 <= 84953 & MedianIncome2021 > 69572 ~ 2,                                          MedianIncome2021 <= 69572 & MedianIncome2021 > 53226 ~ 1,                                          MedianIncome2021 <= 53226 ~ 0))table(ACSdata$median_income_quartile) # 195 classified# rename zip for mergingACSdata <- ACSdata %>%  rename(zip_5dig = `5-digit ZIP Code Tabulation Area` )```# Merge the median_income_quartile variable into the screen_demo file```{r}ACSdata <- ACSdata %>%   dplyr::select(zip_5dig, median_income_quartile)screen_demo <- left_join(screen_demo, ACSdata, by = "zip_5dig")table(screen_demo$median_income_quartile, useNA = "always")screen_demo$median_income_quartile <- factor(screen_demo$median_income_quartile, levels = c(0:3), labels = c("Q1", "Q2", "Q3", "Q4"))```# Limit dataset to those encounters 2019 through 2022 for main analysis```{r}screen_demo_final<- screen_demo %>%  mutate(include = if_else(year > '2018' & year < '2023', 1, 0)) %>%  filter(include == 1)# How many unique women?x = unique(screen_demo_final$Epic_MRN)# table of screening encounters by yeartable(screen_demo_final$year)n <- nrow(screen_demo_final) ntable(screen_demo_final$median_income_quartile, useNA = "always") # 465 missing Median_income_quartile# sixth exclusion nn_ex6 <- nrow(screen_demo) - nrow(screen_demo_final)n_ex6```# Filtering out races other than "White", "Black or African American", "Hispanic", "Asian", those with missing data on age to match numbers with coauthor Waken's time series analysis for table 1```{r}RJnumber <- screen_demo_final %>%  filter(year %in% c(2019, 2020, 2021))  %>% # 86,152  filter(race_col %in% c("non-Hispanic White", "non-Hispanic Black or African-American", "Hispanic", "non-Hispanic Asian")) # 1955n <- nrow(RJnumber)n# RJ exclusion nn_ex6b <- nrow(screen_demo_final) - nrow(RJnumber)n_ex6bRJwomen <- as.data.frame(unique(RJnumber$Epic_MRN))nrow(RJwomen)write.csv(RJnumber, "Time trends 09.19.2021.csv")```# Figure 1. ```{r}Figure <- grViz("digraph flowchart {                  # Set the global graph attributes including fontsize  graph [fontname = Arial, fontsize = 10]    # node definitions with substituted label text  node [fontname = Arial, shape = rectangle, fixedsize = false, width = 0.5]   1 [label = 'EPIC records received for mammography encounters n=923,398']   2 [label = 'EPIC records remaining n = 319,492']  3 [label = 'EPIC records included in age group attrition analysis n = 79,983', style = filled, fillcolor = azure2]  4 [label = 'EPIC records included in race/ethnicity attrition analysis n = 79,257', style = filled, fillcolor = azure2]  5 [label = 'EPIC records included in Bayesian state space models analyses n = 231,385', style = filled, fillcolor = azure2]    m1 [label = 'Excluded records: \\n -161,665 in patients not in catchment area \\n -179,440 not screening mammograms \\n -1,073 non-unique based on patient ID and procedure ID \\n -182,802 not linked to an identifiable mammogram site \\n -4,931 in patients <40 years old \\n -73,995 occurring outside of 2019 to 2022 \\n \\n Additional exclusions for Bayesian state space models analyses: \\n - 86,152 encounters in 2022 \\n -1,955 encounters with race/ethnicity other than \\n NHW, NHB, NHA, or Hispanic']  m2 [label = 'Excluded records: \\n -127 from non-unique patients based on patient ID, year, \\n race/ethnicity category, and zipcode median income quartile \\n -239,259 from patients not screened in 2019, \\n -123 from patients with missing zipcode median household income']  m3 [label = 'Excluded records: \\n -726 from patients with race/ethnicity other than NHW, NHB, NHA, or Hispanic']  node [shape=none, width=0, height=0, label='']  p1 -> 2; p2 -> 3; p1 -> 5; p3 -> 4  {rank=same; p1 -> m1}  {rank=same; p2 -> m2}  {rank=same; p3 -> m3}  edge [dir=none]  1 -> p1; 2 -> p2; 3 -> p3}")Figure %>%  DiagrammeRsvg::export_svg() %>%   charToRaw() %>%   rsvg::rsvg_pdf("/Users/kimjohnson/Library/CloudStorage/Box-Box/A_T Drive - kjohnson/Research/Projects/CoronaVirus/Cancer Services/Revision for mammography only/Mammogram paper/PLOS One submission/Revision/Figure 1.pdf")```# Table 1. Demographics of Encounters```{r}library(table1)render.categorical <- function(x, ...) {    c("", sapply(stats.apply.rounding(stats.default(x)), function(age_cat) with(age_cat,      sprintf("%s (%s%%)", prettyNum(FREQ, big.mark=","), PCT))))}label(RJnumber$age) <- "Age (in years)"label(RJnumber$age_cat) <- "Age group (years)"label(RJnumber$race_col) <- "Race/Ethnicity"label(RJnumber$STATE) <- "Patient's State of Residence"label(RJnumber$median_income_quartile) <- "Median income quartile for zipcode of residence"label(RJnumber$marital) <- "Marital status at screening"table1(~ age + age_cat + race_col +  marital + median_income_quartile + STATE|year,        render.continuous = "Mean (SD)",       render.categorical = render.categorical,       data = RJnumber) # this generates table 1 data for time series# calculate n unique observationsn <- length(unique(screen_demo_final$Epic_MRN))n# generate anonymous table 1 file (single age is not included due to privacy risks)table1_data_TS <- RJnumber %>%  dplyr::select(age_cat, race_col, STATE, median_income_quartile, marital, year)```# FIGURES 2 to 4 in separate code file by coauthor RJ Waken# TABLE 3# Make datasets for race/ethnicity and age category attrition analyses```{r}screen_demo_final2 <- screen_demo_final %>%  dplyr::select(Epic_MRN, year, race_col, median_income_quartile, age_cat) %>%    unique() # 319,365 unique# seventh exclusion nn_ex7 <- nrow(screen_demo_final) - nrow(screen_demo_final2)n_ex7# create dataset with each unique ID in rows and year variables indicating whether they were screened in each year 2019 to 2022MRN_year <- screen_demo_final2 %>%  group_by(Epic_MRN, year) %>% # do not want to group by age_cat because people can change age categories  summarize(count_by_year = n()) %>%  ungroup() %>%  pivot_wider(id_cols = c(Epic_MRN), names_from = year, values_from = count_by_year, values_fn = list(count_by_year = sum), values_fill = list(count_by_year = 0)) %>% # 146,644  filter(`2019` >= 1)  #80,106# eigth exclusion nn_ex8 <-nrow(screen_demo_final2) - nrow(MRN_year)n_ex8for_merge <- screen_demo_final %>%  filter(year == 2019) %>%  arrange(Epic_MRN) %>%   dplyr::select(Epic_MRN, age_cat, race_col, median_income_quartile, marital, STATE) %>%  distinct(Epic_MRN, .keep_all = TRUE)  # this only keeps one record for each individual in 2019 80,106 *if age was included here it would not keep only one record because when there were multiple screens in same year the age sometimes differed by a year# need to get age in for_merge by creating dataframe with age at age first encouunter in 2019 and EPIC_MRNfor_merge2 <- screen_demo_final %>%  filter(year == 2019) %>%  arrange(Epic_MRN) %>%   dplyr::select(Epic_MRN, age) %>%  distinct(Epic_MRN, .keep_all = TRUE)  # this only keeps one record for each individual in 2019 80,106for_merge3 <- left_join(for_merge, for_merge2, by = "Epic_MRN")# checknon_unique_data <- for_merge3 %>%  group_by(Epic_MRN) %>%  filter(n() > 1) # no non-unique records# merge in 2019 age, race and median income quartiles, create variables for analysesMRN_year2 <- left_join(MRN_year, for_merge3,  by= "Epic_MRN") %>%  filter(!is.na(median_income_quartile)) %>% # 79,999   mutate(annual = if_else(`2020` >= 1, 1, 0)) %>% # annual first year after the pandemic   mutate(biennial = if_else(`2020`== 0 & `2021` >= 1, 1, 0)) %>% #  biennial 2nd year after the pandemic    mutate(triennial = if_else(`2020` == 0 & `2021` == 0 & `2022`>= 1, 1, 0)) %>% #  triennial 3rd year after the pandemic   mutate(any_pan = if_else(`2020` >= 1 | `2021` >= 1 | `2022` >=1, 1, 0))   # any screen during pandemic# ninth exclusion nn_ex9 <- nrow(MRN_year) - nrow(MRN_year2)n_ex9nrow(as.data.frame(unique(MRN_year2$Epic_MRN)))# check againnon_unique_data <- MRN_year2 %>%  group_by(Epic_MRN) %>%  filter(n() > 1) # no non-unique data```# Create race dataframe```{r}MRN_year2_race <- MRN_year2 %>%  filter(race_col %in% c("non-Hispanic White", "non-Hispanic Black or African-American", "non-Hispanic Asian", "Hispanic")) # 79,257# tenth exclusion nn_ex10 <- nrow(MRN_year2) - nrow(MRN_year2_race)n_ex10```# Table 1 Age and Race attrition analyses```{r}label(MRN_year2$age_cat) <- "Age group (years)"label(MRN_year2$race_col) <- "Race/Ethnicity"label(MRN_year2$STATE) <- "Patient's State of Residence"label(MRN_year2$median_income_quartile) <- "Median income quartile for zipcode of residence"label(MRN_year2$marital) <- "Marital status at screening"table1(~ age + age_cat + race_col +  marital + median_income_quartile + STATE,        render.continuous = "Mean (SD)",       render.categorical = render.categorical,       data = MRN_year2) # this generates age attrition study populationtable1(~ age + age_cat + race_col +  marital + median_income_quartile + STATE,        render.continuous = "Mean (SD)",       render.categorical = render.categorical,       data = MRN_year2_race) # this generates race attrition study population# check pattern by racesummary_table <- MRN_year2_race %>%  group_by(biennial, annual, triennial, any_pan, race_col) %>%  tally()```# Logistic models for race category```{r}# annualmodel0 <- glm(annual ~ race_col + age_cat + median_income_quartile, family = binomial(link = "logit"), MRN_year2_race)nobs(model0)# without median income quartilemodel0_woMIQ <- glm(annual ~ race_col + age_cat , family = binomial(link = "logit"), MRN_year2_race)nobs(model0_woMIQ)# make dataframe of resultsmodel0df<-as.data.frame(tidy(model0, conf.int = TRUE, conf.level = 0.95, exponentiate = TRUE))model0df$model <- "Annual"# biennialmodel1 <- glm(biennial ~ race_col + age_cat + median_income_quartile, family = binomial(link = "logit"), MRN_year2_race)nobs(model1)# make dataframe of resultsmodel1df<-as.data.frame(tidy(model1, conf.int = TRUE, conf.level = 0.95, exponentiate = TRUE))model1df$model <- "Biennial"# triennialmodel1b <- glm(triennial ~ race_col + age_cat + median_income_quartile, family = binomial(link = "logit"), MRN_year2_race)# make dataframe of resultsmodel1bdf<-as.data.frame(tidy(model1b, conf.int = TRUE, conf.level = 0.95, exponentiate = TRUE))model1bdf$model <- "Triennial"# any screening 2020 to 2022model2 <- glm(any_pan ~ race_col + age_cat + median_income_quartile, family = binomial(link = "logit"), MRN_year2_race)# make dataframe of resultsmodel2df<-as.data.frame(tidy(model2, conf.int = TRUE, conf.level = 0.95, exponentiate = TRUE))model2df$model <- "Any during pandemic"Race_model_results <- rbind(model0df, model1df, model1bdf, model2df)Race_model_results <- Race_model_results %>%  dplyr::select(term, estimate, conf.low, conf.high, p.value, model)rounded_Race_model_results <- Race_model_results %>%  mutate_if(is.numeric, ~round(., 2))```# check N's```{r}table1(~as.factor(annual) + as.factor(biennial) + as.factor(triennial) + as.factor(any_pan) |race_col, data = MRN_year2_race )```# Logistic models for age category```{r}# set 50 to 59 as referenceMRN_year2$age_cat <- factor(MRN_year2$age_cat, levels = c("50-59", "40-49", "60-69", "70-79", "≥0"))# annualmodel0 <- glm(annual ~ age_cat + median_income_quartile, family = binomial(link = "logit"), MRN_year2)summary(model0)# make dataframe of resultsmodel0df_age <- as.data.frame (tidy(model0, conf.int = TRUE, conf.level = 0.95, exponentiate = TRUE))model0df_age$model <- "Annual"# biennialmodel1 <- glm(biennial ~ age_cat + median_income_quartile, family = binomial(link = "logit"), MRN_year2)summary(model1)# make dataframe of resultsmodel1df_age <- as.data.frame (tidy(model1, conf.int = TRUE, conf.level = 0.95, exponentiate = TRUE))model1df_age$model <- "Biennial"# triennialmodel1b <- glm(triennial ~ age_cat + median_income_quartile, family = binomial(link = "logit"), MRN_year2)summary(model1b)# make dataframe of resultsmodel1bdf_age <- as.data.frame (tidy(model1b, conf.int = TRUE, conf.level = 0.95, exponentiate = TRUE))model1bdf_age$model <- "Triennial"# any screening 2020 to 2022model2 <- glm(any_pan ~ age_cat + median_income_quartile, family = binomial(link = "logit"), MRN_year2)summary(model2)# make dataframe of resultsmodel2df_age <- as.data.frame (tidy(model2, conf.int = TRUE, conf.level = 0.95, exponentiate = TRUE))model2df_age$model <- "Any during pandemic"Age_model_results <- rbind(model0df_age, model1df_age, model1bdf_age, model2df_age)Age_model_results <- Age_model_results %>%  dplyr::select(term, estimate, conf.low, conf.high, p.value, model)rounded_Age_model_results <- Age_model_results %>%  mutate_if(is.numeric, ~round(., 2))```# Table 3 sensitivity analysis, create 2018 and 2019 data for an attrition analysis to see if similar pattern of attrition existed prior to the pandemic```{r}# Start from screen_demo that has 2018 obs# use screened in 2018 to determine if they came back in 2019screen_demo_final2_with2018 <- screen_demo %>%  filter(year %in% c(2018, 2019)) %>%  filter(procedure_date > "2018-06-01") %>% # 6/2/2018 is when EPIC was implemented  dplyr::select(Epic_MRN, year, race_col, median_income_quartile, age_cat) %>%    unique() # 119,202 unique# create dataset with each unique ID in rows and year variables indicating whether they were screened in each year 2018MRN_year_sens <- screen_demo_final2_with2018 %>%  group_by(Epic_MRN, year) %>% # do not want to group by age_cat because people can change age categories  summarize(count_by_year = n()) %>%  ungroup() %>%  pivot_wider(id_cols = c(Epic_MRN), names_from = year, values_from = count_by_year, values_fn = list(count_by_year = sum), values_fill = list(count_by_year = 0)) %>%  filter(`2018` >= 1)  # 39,095for_merge <- screen_demo %>%  filter(year == 2018) %>%  arrange(Epic_MRN) %>%   dplyr::select(Epic_MRN, age_cat, race_col, median_income_quartile, marital, STATE) %>%  distinct(Epic_MRN, .keep_all = TRUE)  # this only keeps one record for each individual in 2018 52,226 *if age was included here it would not keep only one record because when there were multiple screens in same year the age sometimes differed by a year# need to get age in for_merge by creating dataframe with age at age first encouunter in 2019 and EPIC_MRNfor_merge2 <- screen_demo %>%  filter(year == 2018) %>%  arrange(Epic_MRN) %>%   dplyr::select(Epic_MRN, age) %>%  distinct(Epic_MRN, .keep_all = TRUE)  # this only keeps one record (first) for each individual in 2019 52,226for_merge3 <- left_join(for_merge, for_merge2, by = "Epic_MRN") #52,226# merge in 2018 age, race and median income quartiles, create variables for analysesMRN_year2_sens <- left_join(MRN_year_sens, for_merge,  by= "Epic_MRN") %>%  filter(!is.na(median_income_quartile)) %>% # 39,035   mutate(annual = if_else(`2019` >= 1, 1, 0)) # annual# Create race dataframeMRN_year2_sens_race <- MRN_year2_sens %>%  filter(race_col %in% c("non-Hispanic White", "non-Hispanic Black or African-American", "non-Hispanic Asian", "Hispanic")) #38,654```# Logistic model age category```{r}# set 50 to 59 as referenceMRN_year2_sens$age_cat <- factor(MRN_year2_sens$age_cat, levels = c("50-59", "40-49", "60-69", "70-79", "≥0"))model1 <- glm(annual ~ age_cat + median_income_quartile, family = binomial(link = "logit"), MRN_year2_sens)# make dataframe of resultsmodel1df_age_sens <- as.data.frame (tidy(model1, conf.int = TRUE, conf.level = 0.95, exponentiate = TRUE))```# Export results```{r}sheets <- list("Logistic results race" = model1df,               "Multinomial results race" = model2df,               "Logistic results age" = model1df_age,               "Multinomial results age" = model2df_age,               "Logistic results sens race" = model1df_race_sens,               "Logistic results sens age" = model1df_age_sens)write.xlsx(sheets, file="Table3.xlsx", showNA=TRUE, overwrite = TRUE)```# Files for Plos One```{r}sheets <- list("Zipcodes" = zipcodes,               "ACSdata" = ACSdata)write.xlsx(sheets, file="datafiles.xlsx", showNA=TRUE, overwrite = TRUE)```
