# Supplementary material for: Impact of COVID-19 pandemic on breast cancer screening in a large midwestern United States academic medical center
Source: PLoS One. 2024 May 20;19(5):e0303280. doi: 10.1371/journal.pone.0303280 (PMC11104587; doi:10.1371/journal.pone.0303280)
Supplement: S3 File — It includes wrangling data from an administrative data format detailing individual instances of claims representing routine screening mammograms to weekly counts by group as well as Bayesian model specification and modeling using Stan. (RTF) [file pone.0303280.s003.rtf]

rm(list = ls())load("new_race_res.RData")load("new_agec_res.RData")load("race_age_data.RData")library(rstan)library(tidyverse)fit_race_summary <- bind_cols(tibble(parname = rownames(summary(fit_race)$summary)),                              summary(fit_race)$summary)fit_race_summary %>%  filter(grepl(pattern = "^ascend_scale", x = parname))fit_race_summary %>%  filter(grepl(pattern = "^ascend_shift", x = parname))fit_race_summary %>%  filter(grepl(pattern = "^descend_scale", x = parname))fit_race_summary %>%  filter(grepl(pattern = "^descend_shift", x = parname))fit_race_summary %>%  filter(grepl(pattern = "alpha", x = parname))get_decay_comps <- function(fitobj, fittable, whichpar) {  nchains <- length(fitobj@sim$samples)    ### lets find the mean of the decay values, use inv var weighting  inv_var_mean <- fittable %>% filter(grepl(pattern = paste0("^", whichpar),                                            x = parname)) %>%    summarize(mean_val = weighted.mean(x = mean, w = 1 / (sd ^ 2)))    ### find p-value that describes if difference from overall mean  sampled_vals <- unlist(sapply(1:nchains,                                function(x) fitobj@sim$samples[[x]][grepl(pattern = paste0("^", whichpar), x = names(fitobj@sim$samples[[x]]))]))    grand_mean <- mean(sampled_vals)  total_var <- var(sampled_vals)    pval <- 2 * min(mean(sampled_vals <= 0), mean(sampled_vals >= 0))  return(pval)}fit_race@sim$samples[[1]]$`ascend_scale0[1]`fit_race@sim$samples[[1]]$`ascend_shift0[1]`race_results_table <- fit_race_summary %>%  tidyr::separate(col = parname, into = c("parname", "indices"), sep = "\\[") %>%  filter(parname %in% c("pred_counts", "np_counts", "pred_diff", "obs_diff", "alpha", "balpha")) %>%  mutate(indices = as.numeric(gsub("\\]", "", indices))) %>%  select(indices, parname, mean, l25 = `2.5%`, u95 = `97.5%`) %>%  pivot_longer(-c(parname, indices)) %>%  pivot_wider(id_cols = c(indices, name), names_from = "parname", values_from = "value") %>%  rename(quantity = name) %>%  pivot_longer(c(alpha, balpha, pred_counts, np_counts, pred_diff, obs_diff),               names_to = "parname") %>%  pivot_wider(names_from = quantity, values_from = value) %>%  mutate(gindex = indices %% 4,         race = factor(gindex,                       levels = 0:3,                       labels = c("A", "W", "B", "H"))) %>%  mutate(rindex = case_when(    gindex == 0 ~ 4,    TRUE ~ gindex  )) %>%  inner_join(race_data %>%               mutate(indices = row_number()))diagnostics_table <- fit_race_summary %>%  tidyr::separate(col = parname, into = c("parname", "indices"), sep = "\\[") %>%  filter(parname %in% c("betas", "betasdrop")) %>%  tidyr::separate(col = indices, into = c("tindex", "k"), sep = ",") %>%  mutate(k = as.numeric(gsub("\\]", "", k)),         tindex = as.numeric(tindex)) %>%  select(tindex, k, parname, mean, l25 = `2.5%`, u95 = `97.5%`) %>%  pivot_longer(-c(parname, tindex, k)) %>%  mutate(parname = paste0(parname, k)) %>%  select(-k) %>%  pivot_wider(id_cols = c(tindex, name), names_from = "parname", values_from = "value") %>%  inner_join(race_data %>%               select(tindex, pandemic, week_of) %>% unique())mu_table <- fit_race_summary %>%  tidyr::separate(col = parname, into = c("parname", "indices"), sep = "\\[") %>%  filter(grepl(pattern = "^mu$", x = parname)) %>%  tidyr::separate(col = indices, into = c("tindex", "rindex"), sep = ",") %>%  mutate(tindex = as.numeric(tindex),         rindex = as.numeric(gsub(pattern = "\\]", replacement = "", x = rindex))) %>%  inner_join(race_data %>%               select(n, groupvar, rindex, tindex, week_of)) %>%  mutate(year = year(week_of))# ggplot(data = mu_table %>%#          mutate(day_of = as.Date("1/1/2020", format = "%m/%d/%Y") + days(yday(week_of) - 1)), #        aes(x = day_of, color = as.factor(year))) +#   geom_point(aes(y = n)) + #   geom_line(aes(y = exp(mean))) + #   facet_wrap(~ groupvar, scales = "free_y") +#   theme(axis.text.x = element_text(angle = 45))race_results_table %>%  filter(parname == "pred_counts") %>%  group_by(groupvar) %>%  summarize(exclusive = mean(n > l25 & n < u95),            inclusive = mean(n >= l25 & n <= u95))race_results_table %>%  filter(grepl(pattern = "alpha$", x = parname)) %>%  filter(pandemic == 1) %>%  print(n = Inf)bind_cols(tibble(parname = rownames(summary(fit_race)$summary)),          summary(fit_race)$summary) %>%  filter(grepl(pattern = "^ascend|^descend", x = parname)) %>%  print(n = Inf)bind_cols(tibble(parname = rownames(summary(fit_race)$summary)),          summary(fit_race)$summary) %>%  filter(grepl(pattern = "^alpha", x = parname)) %>%  separate(col = parname, into = c("parname", "indices"), sep = "\\[") %>%  mutate(indices = as.numeric(gsub(pattern = "\\]", replacement = "", x = indices)),         rindex = indices %% 4,         rindex = case_when(rindex == 0 ~ 4,                            TRUE ~ rindex),         tindex = floor((indices - 1) / 4) + 1) %>%  select(alpha = mean, l25 = `2.5%`, u975 = `97.5%`, tindex, rindex) %>%  inner_join(race_data %>%               filter(pandemic == 1) %>%               select(tindex, week_of, rindex, race = groupvar, n)) %>%  print(n = Inf)bind_cols(tibble(parname = rownames(summary(fit_race)$summary)),          summary(fit_race)$summary) %>%  tidyr::separate(col = parname, into = c("parname", "indices"), sep = "\\[") %>%  filter(grepl(pattern = "^omega", x = parname)) %>%  tidyr::separate(col = indices, into = c("tindex", "k"), sep = ",") %>%  mutate(k = as.numeric(gsub("\\]", "", k)),         tindex = case_when(           parname == "omegadrop" ~ as.numeric(tindex) + race_data_stan$pstart - 1,           TRUE ~ as.numeric(tindex)         )) %>%  select(tindex, k, parname, mean, l25 = `2.5%`, u95 = `97.5%`) %>%  pivot_longer(-c(parname, tindex, k)) %>%  mutate(parname = paste0(parname, k)) %>%  select(-k) %>%  pivot_wider(id_cols = c(tindex, name), names_from = "parname", values_from = "value") %>%  inner_join(race_data %>%               select(tindex, pandemic, week_of) %>% unique()) %>%  filter(!is.na(omegadrop1),         name == "mean")  %>%  print(n = Inf)ggplot(race_results_table %>%         filter(parname == "pred_diff",                pandemic == 1)) +  geom_ribbon(aes(x = week_of, ymin = l25, ymax = u95),              fill = "purple", alpha = 0.5) +  geom_line(data = race_results_table %>%              filter(parname == "obs_diff",                     pandemic == 1),            aes(x = week_of, y = mean), alpha = 0.5) +  facet_wrap(~ groupvar, scales = "free_y")ggplot() +  geom_ribbon(data = race_results_table %>%                filter(parname == "np_counts"),              # pandemic == 1,              # week_of < as.Date("9/1/2020", format = "%m/%d/%Y"),              # week_of > as.Date("2/1/2020", format = "%m/%d/%Y")),              aes(x = week_of, ymin = l25, ymax = u95),              fill = "cyan3") +  geom_ribbon(data = race_results_table %>%                filter(parname == "pred_counts"),              # pandemic == 1,              # week_of < as.Date("9/1/2020", format = "%m/%d/%Y"),              # week_of > as.Date("2/1/2020", format = "%m/%d/%Y")),              aes(x = week_of, ymin = l25, ymax = u95),              fill = "coral2") +  geom_point(data = race_results_table %>%               filter(parname == "pred_counts"),             # pandemic == 1,             # week_of < as.Date("9/1/2020", format = "%m/%d/%Y"),             # week_of > as.Date("2/1/2020", format = "%m/%d/%Y")),             aes(x = week_of, y = n), size = 0.5, alpha = 0.5) +  facet_wrap(~ groupvar, scales = "free_y") +   theme_bw()ggplot(results_table %>%         filter(parname == "np_counts",                pandemic == 1,                week_of < as.Date("8/1/2020", format = "%m/%d/%Y"))) +  geom_ribbon(aes(x = week_of, ymin = l25, ymax = u95),              fill = "purple", alpha = 0.5) +  geom_point(aes(x = week_of, y = n), size = 0.25, alpha = 0.5) +  facet_wrap(~ groupvar, scales = "free_y")alpha_table <- fit_race_summary %>%  tidyr::separate(col = parname, into = c("parname", "indices"), sep = "\\[") %>%  filter(parname %in% c("alpha_mix")) %>%  mutate(indices = as.numeric(gsub("\\]", "", indices)) +            (race_data_stan$pstart - 1) * race_data_stan$gmax) %>%  select(indices, parname, mean, l25 = `2.5%`, u95 = `97.5%`) %>%  pivot_longer(-c(parname, indices)) %>%  pivot_wider(id_cols = c(indices, name), names_from = "parname", values_from = "value") %>%  rename(quantity = name) %>%  pivot_longer(c(alpha_mix),               names_to = "parname") %>%  pivot_wider(names_from = quantity, values_from = value) %>%  mutate(gindex = indices %% 4,         race = factor(gindex,                       levels = 0:3,                       labels = c("A", "W", "B", "H"))) %>%  mutate(rindex = case_when(    gindex == 0 ~ 4,    TRUE ~ gindex  )) %>%  inner_join(race_data %>%               mutate(indices = row_number()))alpha_table %>%  filter(parname == "alpha_mix") %>%  group_by(week_of, groupvar) %>%  summarize(meanci = paste0(round(mean, 3), ", (",                            round(l25, 3), ", ",                            round(u95, 3), ")"),            meanprop = mean) %>%  print(n = Inf)ggplot(data = alpha_table %>%         filter(parname == "alpha_mix")) +   geom_ribbon(aes(x = week_of, ymin = l25, ymax = u95, fill = groupvar),              alpha = 0.5)  results_table %>% filter(grepl(pattern = "alpha$", x = parname),                         pandemic == 1) %>%  print(n = Inf)fit_race_draws <- rstan::extract(fit_race)race_pred_draws <- tibble(pred_counts = fit_race_draws$pred_counts[,(((race_data_stan$pstart - 1) * race_data_stan$gmax)+1):(race_data_stan$pend * race_data_stan$gmax)] %>% as.numeric(),                          np_counts = fit_race_draws$np_counts[,(((race_data_stan$pstart - 1) * race_data_stan$gmax)+1):(race_data_stan$pend * race_data_stan$gmax)] %>% as.numeric(),                          alpha = fit_race_draws$alpha_mix %>% as.numeric())race_pred_draws <- race_pred_draws %>%  mutate(race = rep(c(rep("White", 2000),                      rep("Black or African American", 2000),                      rep("Hispanic", 2000),                      rep("Asian", 2000)),                    22))race_pred_draws %>%  # filter(alpha < 1) %>%  group_by(race) %>%  summarize(totmissed = sum((1 - alpha) * (np_counts - pred_counts)) / 2000,            missrate = weighted.mean((np_counts - pred_counts) / np_counts,                                      w = 1 - alpha),            l025 = DescTools::Quantile((np_counts - pred_counts) / np_counts,                                        weights = 1 - alpha,                                       probs = 0.025),            u975 = DescTools::Quantile((np_counts - pred_counts) / np_counts,                                        weights = 1 - alpha,                                       probs = 0.975)) %>%  print(n = Inf)get_decay_comps <- function(fitobj = fit_race, fittable = fit_race_summary) {  ### lets find the mean of the decay values, use inv var weighting  inv_var_mean_scale <- fittable %>% filter(grepl(pattern = "^ascend_scale",                                            x = parname)) %>%    summarize(mean_val = weighted.mean(x = mean, w = 1 / (sd ^ 2)))    ### lets find the mean of the decay values, use inv var weighting  inv_var_mean_shift <- fittable %>% filter(grepl(pattern = "^ascend_shift",                                                  x = parname)) %>%    summarize(mean_val = weighted.mean(x = mean, w = 1 / (sd ^ 2)))    ### find p-value that describes if difference from overall mean  all_draws <- rstan::extract(fitobj)    sampled_vals_scale <- as_tibble(all_draws$ascend_scale0) %>%    pivot_longer(everything(), names_to = "group", values_to = "scale") %>%    mutate(scale = scale - as.numeric(inv_var_mean_scale))  sampled_vals_shift <- as_tibble(all_draws$ascend_shift0) %>%    pivot_longer(everything(), names_to = "group", values_to = "shift") %>%    mutate(shift = shift - as.numeric(inv_var_mean_shift))    samp_vals <- bind_cols(sampled_vals_scale,                          sampled_vals_shift %>% select(-group)) %>%    pivot_longer(-group, names_to = "quan") %>%    group_by(group, quan) %>%    summarize(outval = (mean(value) / sd(value))^2) %>%    ungroup()    pval <- samp_vals %>%    ungroup() %>%    summarize(sum(outval)) %>%    unlist() %>%    pchisq(.,            df = nrow(samp_vals),           lower.tail = FALSE)    return(pval)}fit_agec_summary <- bind_cols(tibble(parname = rownames(summary(fit_agec)$summary)),                              summary(fit_agec)$summary)get_decay_comps(fit_race, fit_race_summary)get_decay_comps(fit_agec, fit_agec_summary)get_undecay_comps <- function(fitobj = fit_race, fittable = fit_race_summary) {  ### lets find the mean of the decay values, use inv var weighting  inv_var_mean_scale <- fittable %>% filter(grepl(pattern = "^descend_scale",                                                  x = parname)) %>%    summarize(mean_val = weighted.mean(x = mean, w = 1 / (sd ^ 2)))    ### lets find the mean of the decay values, use inv var weighting  inv_var_mean_shift <- fittable %>% filter(grepl(pattern = "^descend_shift",                                                  x = parname)) %>%    summarize(mean_val = weighted.mean(x = mean, w = 1 / (sd ^ 2)))    ### find p-value that describes if difference from overall mean  all_draws <- rstan::extract(fitobj)    sampled_vals_scale <- as_tibble(all_draws$ascend_scale0) %>%    pivot_longer(everything(), names_to = "group", values_to = "scale") %>%    mutate(scale = scale - as.numeric(inv_var_mean_scale))  sampled_vals_shift <- as_tibble(all_draws$ascend_shift0) %>%    pivot_longer(everything(), names_to = "group", values_to = "shift") %>%    mutate(shift = shift - as.numeric(inv_var_mean_shift))    samp_vals <- bind_cols(sampled_vals_scale,                          sampled_vals_shift %>% select(-group)) %>%    pivot_longer(-group, names_to = "quan") %>%    group_by(group, quan) %>%    summarize(outval = (mean(value) / sd(value))^2) %>%    ungroup()    pval <- samp_vals %>%    ungroup() %>%    summarize(sum(outval)) %>%    unlist() %>%    pchisq(.,            df = nrow(samp_vals),           lower.tail = FALSE)    return(pval)}get_undecay_comps(fit_race, fit_race_summary)get_undecay_comps(fit_agec, fit_agec_summary)
