# Supplementary material for: Impact of COVID-19 pandemic on breast cancer screening in a large midwestern United States academic medical center
Source: PLoS One. 2024 May 20;19(5):e0303280. doi: 10.1371/journal.pone.0303280 (PMC11104587; doi:10.1371/journal.pone.0303280)
Supplement: S4 File — (RTF) [file pone.0303280.s004.rtf]

---title: "COVID screening plots and results"author: "Caitlin, Kim, and RJ"date: "2023-09-20"output: html_document---  ```{r setup, include=FALSE}library(rstan)library(tidyverse)library(lubridate)load("race_age_data.RData")load("new_race_res.RData")load("new_agec_res.RData")race_data <- race_data %>%  mutate(holiday = factor(holiday,                          levels = 0:1,                          labels = c("no", "yes")),         )agec_data <- agec_data %>%  mutate(holiday = factor(holiday,                          levels = 0:1,                          labels = c("no", "yes")))ever_data <- ever_data %>%  mutate(holiday = factor(holiday,                          levels = 0:1,                          labels = c("no", "yes")))my_date_format <- function(){   function(x)   {       m <- format(x,"%b")       y <- format(x,"%Y")       ifelse(duplicated(y),m,paste(y,m))   }}```## ResultsWe're going to look at results within each of the groupings that we have, starting with race, then age group. In each section, we'll look at- model performance- drop in visits at height of pandemic- when was the pandemic affecting screenings?  - mean of delayed or missed visits over time- total number of delayed or missed visits- rate of delayed or missed visits relative to expected### Race#### Model performanceThe below shows the model fit (the line), the observations (the dots), and the 95% CIs for predicted observations (the bands). I have also included the prediction bands (cyan) for the model where no pandemic occured - the difference between this and the modeled process during the pandemic is what yields the "missed visits" numbers.```{r, echo = FALSE, message = FALSE, warning = FALSE}fit_race_summary <- bind_cols(tibble(parname = rownames(summary(fit_race)$summary)),                              summary(fit_race)$summary)race_results_table <- fit_race_summary %>%  tidyr::separate(col = parname, into = c("parname", "indices"), sep = "\\[") %>%  filter(parname %in% c("pred_counts", "np_counts", "pred_diff", "obs_diff", "alpha", "balpha")) %>%  mutate(indices = as.numeric(gsub("\\]", "", indices))) %>%  select(indices, parname, mean, l25 = `2.5%`, u975 = `97.5%`) %>%  pivot_longer(-c(parname, indices)) %>%  pivot_wider(id_cols = c(indices, name), names_from = "parname", values_from = "value") %>%  rename(quantity = name) %>%  pivot_longer(c(alpha, balpha, pred_counts, np_counts, pred_diff, obs_diff),               names_to = "parname") %>%  pivot_wider(names_from = quantity, values_from = value) %>%  mutate(gindex = indices %% 4,         race = factor(gindex,                       levels = 0:3,                       labels = c("A", "W", "B", "H"))) %>%  mutate(rindex = case_when(    gindex == 0 ~ 4,    TRUE ~ gindex  )) %>%  inner_join(race_data %>%               mutate(indices = row_number()))race_results_table <- race_results_table %>%  mutate(groupvar = factor(groupvar,                           levels = c("White",                                       "Black or African American",                                      "Hispanic",                                      "Asian"),                           labels = c("NH White",                                       "NH Black or African American",                                      "Hispanic",                                      "NH Asian")))race_count_plot <- ggplot() +  geom_ribbon(data = race_results_table %>%                filter(parname == "np_counts"),              # pandemic == 1,              # week_of < as.Date("9/1/2020", format = "%m/%d/%Y"),              # week_of > as.Date("2/1/2020", format = "%m/%d/%Y")),              aes(x = week_of, ymin = l25, ymax = u975),              fill = "cyan3") +  geom_line(data = race_results_table %>%                filter(parname == "np_counts"),              # pandemic == 1,              # week_of < as.Date("9/1/2020", format = "%m/%d/%Y"),              # week_of > as.Date("2/1/2020", format = "%m/%d/%Y")),              aes(x = week_of, y = mean),              color = "cyan4") +  geom_ribbon(data = race_results_table %>%                filter(parname == "pred_counts"),              # pandemic == 1,              # week_of < as.Date("9/1/2020", format = "%m/%d/%Y"),              # week_of > as.Date("2/1/2020", format = "%m/%d/%Y")),              aes(x = week_of, ymin = l25, ymax = u975),              fill = "coral2") +  geom_line(data = race_results_table %>%                filter(parname == "pred_counts"),              # pandemic == 1,              # week_of < as.Date("9/1/2020", format = "%m/%d/%Y"),              # week_of > as.Date("2/1/2020", format = "%m/%d/%Y")),              aes(x = week_of, y = mean),              color = "coral4") +  geom_point(data = race_results_table %>%               filter(parname == "pred_counts"),             # pandemic == 1,             # week_of < as.Date("9/1/2020", format = "%m/%d/%Y"),             # week_of > as.Date("2/1/2020", format = "%m/%d/%Y")),             aes(x = week_of, y = n, shape = holiday), alpha = 0.5, size = 0.75) +  # facet_wrap(~ groupvar, scales = "free_y") +   facet_wrap(~ groupvar, scales = "free_y", ncol = 1) +   theme_bw() +   scale_x_date(date_breaks = "1 month", name = "Week",               labels = my_date_format(),               limits = as.Date(c('12/31/2018', '1/1/2022'), format="%d/%m/%Y"),               expand = c(1 / 365, 1 / 365)) +  theme(legend.position = "bottom",        axis.text.x = element_text(angle = 90,                                    vjust = 0.2,                                   hjust = 0.95)) +   ylab("Mean number count of screenings")ggsave(race_count_plot, device = "pdf", filename = "race_count_plot.pdf", height = 11, width = 8.5, units = "in")race_count_plot```We can see that the model does a good job of characterizing the seasonal trend and capturing what's happening in the different groups. We do see an uptick in visits post pandemic, which is being described by the time-varying group mean parameters.We also should assess coverage, or the proportion of times a prediction interval contains the observed value, to make sure that our model is doing a good job of characterizing what's happening. Note a few things:  - predictions are "current time", not "future time", so this is not the same as forecasting- I used two definitions for coverage using layperson's language since, for most frequentist analyses, predictions are based on means which take on non-discrete values, and in Bayesian analyses, posterior predictive distributions are generated via MCMC draws and thus are comprised of distributions of discrete counts  - exclusive means "the observed values are less than the upper 97.5th quantile and greater than the lower 2.5th quantile of the posterior predictive MCMC draws"  - inclusive means "the observed values are less than *or equal to* the upper 97.5th quantile and greater than *or equal to* the lower 2.5th quantile of the posterior predictive MCMC draws"  - no one really uses these terms to describe coverage, but it's a useful exercise in investigating overcoverage. Inclusive is what we'll report - but also tends to over cover with discrete distributions. I like to look at both in that case as a check.```{r, echo = FALSE}knitr::kable(  race_results_table %>%  filter(parname == "pred_counts") %>%  group_by(groupvar) %>%  summarize(exclusive = mean(n > l25 & n < u975),            inclusive = mean(n >= l25 & n <= u975)),  digits = 4)```We tend to overcover in all groups. The fact that the "exclusive" coverage values are lower than 95\% is encouraging in that it leads me to believe that we aren't just specifying a variance that's too big.- as a side note, this variance/spread issue tends to be more of a problem when you have some kind of parameter that describes kurtosis or heavy tailed-ness (like the degrees of freedom parameter $\nu$ in Student's t, or the additional parameter in the negative binomial distribution). The model is incentivized to use these parameters to "soak up" a lot of variability, and you end up with something that's not particularly generalizable and a little wacky looking. It just attributes all variance to the tails and produces massive interval estimates everywhere. There's an art to taming that (that we do not want to get into unless we have to)#### Drop in visits at height of pandemicWe look at the "drop in visits" based on - the model's belief that the pandemic was affecting the process, and- the difference in the number of predicted visits without the pandemic (cyan bands in the above figure) and the predicted visits during the pandemic.```{r, echo = FALSE, message = FALSE, warning = FALSE}fit_race_draws <- rstan::extract(fit_race)race_pred_draws <- tibble(pred_counts = fit_race_draws$pred_counts[,(((race_data_stan$pstart - 1) * race_data_stan$gmax)+1):(race_data_stan$pend * race_data_stan$gmax)] %>% as.numeric(),                          np_counts = fit_race_draws$np_counts[,(((race_data_stan$pstart - 1) * race_data_stan$gmax)+1):(race_data_stan$pend * race_data_stan$gmax)] %>% as.numeric(),                          alpha = fit_race_draws$alpha_mix %>% as.numeric())race_pred_draws <- race_pred_draws %>%  mutate(race = rep(c(rep("White", 2000),                      rep("Black or African American", 2000),                      rep("Hispanic", 2000),                      rep("Asian", 2000)),                    22))knitr::kable(  race_pred_draws %>%  # filter(alpha < 1) %>%  group_by(race) %>%  summarize(totmissed = sum((1 - alpha) * (np_counts - pred_counts)) / 2000,            missrate = weighted.mean((np_counts - pred_counts) / np_counts,                                      w = 1 - alpha),            l025 = DescTools::Quantile((np_counts - pred_counts) / np_counts,                                        weights = 1 - alpha,                                       probs = 0.025),            u975 = DescTools::Quantile((np_counts - pred_counts) / np_counts,                                        weights = 1 - alpha,                                       probs = 0.975)),  digits = 4)```#### When was the pandemic affecting screenings?Here, we look at the minimum and maximum dates where the lower 2.5th percentile of MCMC draws for the the pandemic effect parameter is at least 0.1. We see```{r, echo = FALSE, warning=FALSE, message = FALSE}race_alpha_table <- fit_race_summary %>%  tidyr::separate(col = parname, into = c("parname", "indices"), sep = "\\[") %>%  filter(parname %in% c("alpha_mix")) %>%  mutate(indices = as.numeric(gsub("\\]", "", indices)) +            (race_data_stan$pstart - 1) * race_data_stan$gmax) %>%  select(indices, parname, mean, l25 = `2.5%`, u975 = `97.5%`) %>%  pivot_longer(-c(parname, indices)) %>%  pivot_wider(id_cols = c(indices, name), names_from = "parname", values_from = "value") %>%  rename(quantity = name) %>%  pivot_longer(c(alpha_mix),               names_to = "parname") %>%  pivot_wider(names_from = quantity, values_from = value) %>%  mutate(gindex = indices %% 4,         race = factor(gindex,                       levels = 0:3,                       labels = c("A", "W", "B", "H"))) %>%  mutate(rindex = case_when(    gindex == 0 ~ 4,    TRUE ~ gindex  )) %>%  inner_join(race_data %>%               mutate(indices = row_number()))knitr::kable(  race_alpha_table %>%  filter(l25 < 0.9) %>%  group_by(groupvar) %>%  summarize(first_week = min(week_of),            last_week = max(week_of)))```#### Mean of delayed or missed visits over time in 2020Here's a plot to show the mean and confidence interval for the predicted drop in visits within group:  ```{r, echo = FALSE, warning = FALSE, message = FALSE}race_preddrop_plot <- ggplot(race_results_table %>%         filter(parname == "pred_diff",                pandemic == 1)) +  geom_ribbon(aes(x = week_of, ymin = l25, ymax = u975),              fill = "purple", alpha = 0.5) +  geom_line(data = race_results_table %>%              filter(parname == "obs_diff",                     pandemic == 1),            aes(x = week_of, y = mean), alpha = 0.5) +  facet_wrap(~ groupvar, scales = "free_y") +  theme_bw() +   ylab("Predicted drop in visits") +   xlab("Week")ggsave(race_preddrop_plot, device = "pdf", filename = "race_preddrop.pdf", height = 8.5, width = 11, units = "in")race_preddrop_plot```#### Did the groups delay or miss visits at the different rates?In order to formally test how each group returned to regular screenings, we test for differences in the part of the model that accounts for differences in the "return to normalcy." This is effectively a test of how the parameters controlling that return to normalcy differ from the mean across all groups. For race, we see a p-value of:  ```{r, echo = FALSE, warning = FALSE, message = FALSE}### new decay codeget_decay_comps <- function(fitobj = fit_race, fittable = fit_race_summary) {  ### lets find the mean of the decay values, use inv var weighting  inv_var_mean_scale <- fittable %>% filter(grepl(pattern = "^ascend_scale",                                                  x = parname)) %>%    summarize(mean_val = weighted.mean(x = mean, w = 1 / (sd ^ 2)))    ### lets find the mean of the decay values, use inv var weighting  inv_var_mean_shift <- fittable %>% filter(grepl(pattern = "^ascend_shift",                                                  x = parname)) %>%    summarize(mean_val = weighted.mean(x = mean, w = 1 / (sd ^ 2)))    ### find p-value that describes if difference from overall mean  all_draws <- rstan::extract(fitobj)    sampled_vals_scale <- as_tibble(all_draws$ascend_scale0) %>%    pivot_longer(everything(), names_to = "group", values_to = "scale") %>%    mutate(scale = scale - as.numeric(inv_var_mean_scale))  sampled_vals_shift <- as_tibble(all_draws$ascend_shift0) %>%    pivot_longer(everything(), names_to = "group", values_to = "shift") %>%    mutate(shift = shift - as.numeric(inv_var_mean_shift))    samp_vals <- bind_cols(sampled_vals_scale,                          sampled_vals_shift %>% select(-group)) %>%    pivot_longer(-group, names_to = "quan") %>%    group_by(group, quan) %>%    summarize(outval = (mean(value) / sd(value))^2) %>%    ungroup()    pval <- samp_vals %>%    ungroup() %>%    summarize(`p-value` = sum(outval)) %>%    unlist() %>%    pchisq(.,            df = nrow(samp_vals),           lower.tail = FALSE)    return(pval)}get_decay_comps(fit_race, fit_race_summary)```### Age groups#### Model performanceThe below shows the model fit (the line), the observations (the dots), and the 95% CIs for predicted observations (the bands). I have also included the prediction bands (cyan) for the model where no pandemic occured - the difference between this and the modeled process during the pandemic is what yields the "missed visits" numbers.```{r, echo = FALSE, message = FALSE, warning = FALSE}fit_agec_summary <- bind_cols(tibble(parname = rownames(summary(fit_agec)$summary)),                              summary(fit_agec)$summary)agec_results_table <- fit_agec_summary %>%  tidyr::separate(col = parname, into = c("parname", "indices"), sep = "\\[") %>%  filter(parname %in% c("pred_counts", "np_counts", "pred_diff", "obs_diff", "alpha", "balpha")) %>%  mutate(indices = as.numeric(gsub("\\]", "", indices))) %>%  select(indices, parname, mean, l25 = `2.5%`, u975 = `97.5%`) %>%  pivot_longer(-c(parname, indices)) %>%  pivot_wider(id_cols = c(indices, name), names_from = "parname", values_from = "value") %>%  rename(quantity = name) %>%  pivot_longer(c(alpha, balpha, pred_counts, np_counts, pred_diff, obs_diff),               names_to = "parname") %>%  pivot_wider(names_from = quantity, values_from = value) %>%  mutate(gindex = indices %% 5) %>%  mutate(rindex = case_when(    gindex == 0 ~ 5,    TRUE ~ gindex  )) %>%  inner_join(agec_data %>%               mutate(indices = row_number()))age_count_plot <- ggplot() +  geom_ribbon(data = agec_results_table %>%                filter(parname == "np_counts"),              # pandemic == 1,              # week_of < as.Date("9/1/2020", format = "%m/%d/%Y"),              # week_of > as.Date("2/1/2020", format = "%m/%d/%Y")),              aes(x = week_of, ymin = l25, ymax = u975),              fill = "cyan3") +  geom_line(data = agec_results_table %>%                filter(parname == "np_counts"),              # pandemic == 1,              # week_of < as.Date("9/1/2020", format = "%m/%d/%Y"),              # week_of > as.Date("2/1/2020", format = "%m/%d/%Y")),              aes(x = week_of, y = mean),              color = "cyan4") +  geom_ribbon(data = agec_results_table %>%                filter(parname == "pred_counts"),              # pandemic == 1,              # week_of < as.Date("9/1/2020", format = "%m/%d/%Y"),              # week_of > as.Date("2/1/2020", format = "%m/%d/%Y")),              aes(x = week_of, ymin = l25, ymax = u975),              fill = "coral2") +  geom_line(data = agec_results_table %>%                filter(parname == "pred_counts"),              # pandemic == 1,              # week_of < as.Date("9/1/2020", format = "%m/%d/%Y"),              # week_of > as.Date("2/1/2020", format = "%m/%d/%Y")),              aes(x = week_of, y = mean),              color = "coral4") +  geom_point(data = agec_results_table %>%               filter(parname == "pred_counts"),             # pandemic == 1,             # week_of < as.Date("9/1/2020", format = "%m/%d/%Y"),             # week_of > as.Date("2/1/2020", format = "%m/%d/%Y")),             aes(x = week_of, y = n, shape = holiday), alpha = 0.5, size = 0.75) +  # facet_wrap(~ groupvar, scales = "free_y") +   facet_wrap(~ groupvar, scales = "free_y", ncol = 1) +   theme_bw() +   scale_x_date(date_breaks = "1 month", name = "Week",               labels = my_date_format(),               limits = as.Date(c('12/31/2018', '1/1/2022'), format="%d/%m/%Y"),               expand = c(1 / 365, 1 / 365)) +  theme(legend.position = "bottom",        axis.text.x = element_text(angle = 90,                                    vjust = 0.2,                                   hjust = 0.95)) +   ylab("Mean number count of screenings")ggsave(age_count_plot, device = "pdf", filename = "age_count_plot.pdf", height = 11, width = 8.5, units = "in")age_count_plot```We can see that the model does a good job of characterizing the seasonal trend and capturing what's happening in the different groups. We do see an uptick in visits post pandemic, which is being described by the time-varying group mean parameters.We also should assess coverage, or the proportion of times a prediction interval contains the observed value, to make sure that our model is doing a good job of characterizing what's happening. Note a few things:  - predictions are "current time", not "future time", so this is not the same as forecasting- I used two definitions for coverage using layperson's language since, for most frequentist analyses, predictions are based on means which take on non-discrete values, and in Bayesian analyses, posterior predictive distributions are generated via MCMC draws and thus are comprised of distributions of discrete counts  - exclusive means "the observed values are less than the upper 97.5th quantile and greater than the lower 2.5th quantile of the posterior predictive MCMC draws"  - inclusive means "the observed values are less than *or equal to* the upper 97.5th quantile and greater than *or equal to* the lower 2.5th quantile of the posterior predictive MCMC draws"  Inclusive is most appropriate - but also tends to over cover with discrete distributions. I like to look at both.```{r, echo = FALSE}knitr::kable(  agec_results_table %>%    filter(parname == "pred_counts") %>%    group_by(groupvar) %>%    summarize(exclusive = mean(n > l25 & n < u975),              inclusive = mean(n >= l25 & n <= u975)),  digits = 4)```We tend to overcover in all groups. The fact that the "exclusive" coverage values are closer to 95\% is encouraging in that it leads me to believe that we aren't just specifying a varaince that's too big.#### Drop in visits at height of pandemicWe look at the "drop in visits" based on - the model's belief that the pandemic was affecting the process, and- the difference in the number of predicted visits without the pandemic (cyan bands in the above figure) and the predicted visits during the pandemic.```{r, echo = FALSE, message = FALSE, warning = FALSE}fit_agec_draws <- rstan::extract(fit_agec)agec_pred_draws <- tibble(pred_counts = fit_agec_draws$pred_counts[,(((agec_data_stan$pstart - 1) * agec_data_stan$gmax)+1):(agec_data_stan$pend * agec_data_stan$gmax)] %>% as.numeric(),                          np_counts = fit_agec_draws$np_counts[,(((agec_data_stan$pstart - 1) * agec_data_stan$gmax)+1):(agec_data_stan$pend * agec_data_stan$gmax)] %>% as.numeric(),                          alpha = fit_agec_draws$alpha_mix %>% as.numeric())agec_pred_draws <- agec_pred_draws %>%  mutate(agec = rep(c(rep("40-49", 2000),                      rep("50-59", 2000),                      rep("60-69", 2000),                      rep("70-79", 2000),                      rep("80+", 2000)),                    22))knitr::kable(  agec_pred_draws %>%    # filter(alpha < 1) %>%    group_by(agec) %>%    summarize(totmissed = sum((1 - alpha) * (np_counts - pred_counts)) / 2000,              missrate = weighted.mean((np_counts - pred_counts) / np_counts,                                        w = 1 - alpha),              l025 = DescTools::Quantile((np_counts - pred_counts) / np_counts,                                          weights = 1 - alpha,                                         probs = 0.025),              u975 = DescTools::Quantile((np_counts - pred_counts) / np_counts,                                          weights = 1 - alpha,                                         probs = 0.975)),  digits = 4)```#### When was the pandemic affecting screenings?Here, we look at the minimum and maximum dates where the lower 2.5th percentile of MCMC draws for the the pandemic effect parameter is at least 0.1 - this is a one sided confidence interval. We see```{r, echo = FALSE, warning=FALSE, message = FALSE}agec_alpha_table <- fit_agec_summary %>%  tidyr::separate(col = parname, into = c("parname", "indices"), sep = "\\[") %>%  filter(parname %in% c("alpha_mix")) %>%  mutate(indices = as.numeric(gsub("\\]", "", indices)) +            (agec_data_stan$pstart - 1) * agec_data_stan$gmax) %>%  select(indices, parname, mean, l25 = `2.5%`, u975 = `97.5%`) %>%  pivot_longer(-c(parname, indices)) %>%  pivot_wider(id_cols = c(indices, name), names_from = "parname", values_from = "value") %>%  rename(quantity = name) %>%  pivot_longer(c(alpha_mix),               names_to = "parname") %>%  pivot_wider(names_from = quantity, values_from = value) %>%  mutate(gindex = indices %% 5) %>%  mutate(rindex = case_when(    gindex == 0 ~ 5,    TRUE ~ gindex  )) %>%  inner_join(agec_data %>%               mutate(indices = row_number()))knitr::kable(  agec_alpha_table %>%    filter(l25 < 0.9) %>%    group_by(groupvar) %>%    summarize(first_week = min(week_of),              last_week = max(week_of)))```#### Mean of delayed or missed visits over time in 2020Here's a plot to show the mean and confidence interval for the predicted drop in visits within group:```{r, echo = FALSE, warning = FALSE, message = FALSE}age_preddrop_plot <- ggplot(agec_results_table %>%         filter(parname == "pred_diff",                pandemic == 1)) +  geom_ribbon(aes(x = week_of, ymin = l25, ymax = u975),              fill = "purple", alpha = 0.5) +  geom_line(data = agec_results_table %>%              filter(parname == "obs_diff",                     pandemic == 1),            aes(x = week_of, y = mean), alpha = 0.5) +  facet_wrap(~ groupvar, scales = "free_y") +  theme_bw() +   ylab("Predicted drop in visits") +   xlab("Week")ggsave(age_preddrop_plot, device = "pdf", filename = "age_preddrop.pdf", height = 8.5, width = 11, units = "in")age_preddrop_plot```#### Did the groups delay or miss visits at the different rates?In order to formally test how each group returned to regular screenings, we test for differences in the part of the model that accounts for differences in the "return to normalcy." This is effectively a test of how the parameters controlling that return to normalcy differ from the mean across all groups. For age groups, we see a p-value of:```{r, echo = FALSE, warning = FALSE, message = FALSE}### new decay codeget_decay_comps(fit_agec, fit_agec_summary)```### Overall#### Model performance```{r, echo = FALSE, message = FALSE, warning = FALSE}load("new_ever_res.RData")fit_ever_summary <- bind_cols(tibble(parname = rownames(summary(fit_ever)$summary)),                              summary(fit_ever)$summary)ever_results_table <- fit_ever_summary %>%  tidyr::separate(col = parname, into = c("parname", "indices"), sep = "\\[") %>%  filter(parname %in% c("pred_counts", "np_counts", "pred_diff", "obs_diff", "alpha", "balpha")) %>%  mutate(indices = as.numeric(gsub("\\]", "", indices))) %>%  select(indices, parname, mean, l25 = `2.5%`, u975 = `97.5%`) %>%  pivot_longer(-c(parname, indices)) %>%  pivot_wider(id_cols = c(indices, name), names_from = "parname", values_from = "value") %>%  rename(quantity = name) %>%  pivot_longer(c(alpha, balpha, pred_counts, np_counts, pred_diff, obs_diff),               names_to = "parname") %>%  pivot_wider(names_from = quantity, values_from = value) %>%  inner_join(ever_data %>%               mutate(indices = row_number()))all_count_plot <- ggplot() +  geom_ribbon(data = ever_results_table %>%                filter(parname == "np_counts"),              # pandemic == 1,              # week_of < as.Date("9/1/2020", format = "%m/%d/%Y"),              # week_of > as.Date("2/1/2020", format = "%m/%d/%Y")),              aes(x = week_of, ymin = l25, ymax = u975),              fill = "cyan3") +  geom_line(data = ever_results_table %>%                filter(parname == "np_counts"),              # pandemic == 1,              # week_of < as.Date("9/1/2020", format = "%m/%d/%Y"),              # week_of > as.Date("2/1/2020", format = "%m/%d/%Y")),              aes(x = week_of, y = mean),              color = "cyan4") +  geom_ribbon(data = ever_results_table %>%                filter(parname == "pred_counts"),              # pandemic == 1,              # week_of < as.Date("9/1/2020", format = "%m/%d/%Y"),              # week_of > as.Date("2/1/2020", format = "%m/%d/%Y")),              aes(x = week_of, ymin = l25, ymax = u975),              fill = "coral2") +  geom_line(data = ever_results_table %>%                filter(parname == "pred_counts"),              # pandemic == 1,              # week_of < as.Date("9/1/2020", format = "%m/%d/%Y"),              # week_of > as.Date("2/1/2020", format = "%m/%d/%Y")),              aes(x = week_of, y = mean),              color = "coral4") +  geom_point(data = ever_results_table %>%               filter(parname == "pred_counts"),             # pandemic == 1,             # week_of < as.Date("9/1/2020", format = "%m/%d/%Y"),             # week_of > as.Date("2/1/2020", format = "%m/%d/%Y")),             aes(x = week_of, y = n, shape = holiday) ,alpha = 0.5) +  # facet_wrap(~ groupvar, scales = "free_y") +   theme_bw() +   scale_x_date(date_breaks = "1 month", name = "Week",               labels = my_date_format(),               limits = as.Date(c('12/31/2018', '1/1/2022'), format="%d/%m/%Y"),               expand = c(1 / 365, 1 / 365)) +  theme(legend.position = "bottom",        axis.text.x = element_text(angle = 90,                                    vjust = 0.2,                                   hjust = 0.95)) +   ylab("Mean number count\nof screenings")ggsave(all_count_plot, device = "pdf", filename = "all_count_plot.pdf", height = 3, width = 8.5, units = "in")all_count_plot```As we can see, the model fits the data quite well. We do see that the predicted "Brownian bridge" seems to *really* drop toward the end of its run - however, that's just a holiday, so the drop is totally expected (even if it looks a little funny). Encouraging that the model was reasonably successful in sorting that out, as that's probably the hardest decision point it has in this entire analysis. If we look at coverage, we see:```{r, echo = FALSE, warning = FALSE, message = FALSE}knitr::kable(  ever_results_table %>%    filter(parname == "pred_counts") %>%    # group_by(groupvar) %>%    summarize(exclusive = mean(n > l25 & n < u975),              inclusive = mean(n >= l25 & n <= u975)),  digits = 4)```#### Drop in visits at height of pandemicIf we want to characterize the difference, we can look at the proportion drop while the pandemic was in effect. Here we see:```{r, echo = FALSE, warning = FALSE, message = FALSE}fit_ever_draws <- rstan::extract(fit_ever)ever_pred_draws <- tibble(pred_counts = fit_ever_draws$pred_counts[,(((ever_data_stan$pstart - 1) * ever_data_stan$gmax)+1):(ever_data_stan$pend * ever_data_stan$gmax)] %>% as.numeric(),                          np_counts = fit_ever_draws$np_counts[,(((ever_data_stan$pstart - 1) * ever_data_stan$gmax)+1):(ever_data_stan$pend * ever_data_stan$gmax)] %>% as.numeric(),                          alpha = fit_ever_draws$alpha_mix %>% as.numeric())ever_pred_draws <- ever_pred_draws %>%  mutate(ever = rep(c(rep("everyone", 2000)), 22))knitr::kable(  ever_pred_draws %>%    # filter(alpha < 1) %>%    # group_by(ever) %>%    summarize(totmissed = sum((1 - alpha) * (np_counts - pred_counts)) / 2000,              missrate = weighted.mean((np_counts - pred_counts) / np_counts,                                        w = 1 - alpha),              l025 = DescTools::Quantile((np_counts - pred_counts) / np_counts,                                          weights = 1 - alpha,                                         probs = 0.025),              u975 = DescTools::Quantile((np_counts - pred_counts) / np_counts,                                          weights = 1 - alpha,                                         probs = 0.975)),  digits = 4)```#### When was the pandemic affecting screenings?Here, we look at the minimum and maximum dates where the upper 95th percentile of MCMC draws for the the pandemic effect parameter is at least 0.1 - this is a one sided confidence interval. We see```{r, echo = FALSE, warning=FALSE, message = FALSE}ever_alpha_table <- fit_race_summary %>%  tidyr::separate(col = parname, into = c("parname", "indices"), sep = "\\[") %>%  filter(parname %in% c("alpha_mix")) %>%  mutate(indices = as.numeric(gsub("\\]", "", indices)) +            (race_data_stan$pstart - 1) * race_data_stan$gmax) %>%  select(indices, parname, mean, l25 = `2.5%`, u975 = `97.5%`) %>%  pivot_longer(-c(parname, indices)) %>%  pivot_wider(id_cols = c(indices, name), names_from = "parname", values_from = "value") %>%  rename(quantity = name) %>%  pivot_longer(c(alpha_mix),               names_to = "parname") %>%  pivot_wider(names_from = quantity, values_from = value) %>%  inner_join(race_data %>%               mutate(indices = row_number()))knitr::kable(  ever_alpha_table %>%  filter(l25 < 0.9) %>%  # group_by(groupvar) %>%  summarize(first_week = min(week_of),            last_week = max(week_of)))```#### Mean of delayed or missed visits over time in 2020Here's a plot to show the mean and confidence interval for the predicted drop in visits:  ```{r, echo = FALSE, warning = FALSE, message = FALSE}all_preddrop_plot <- ggplot(ever_results_table %>%         filter(parname == "pred_diff",                pandemic == 1)) +  geom_ribbon(aes(x = week_of, ymin = l25, ymax = u975),              fill = "purple", alpha = 0.5) +  geom_line(data = ever_results_table %>%              filter(parname == "obs_diff",                     pandemic == 1),            aes(x = week_of, y = mean), alpha = 0.5) +  # facet_wrap(~ groupvar, scales = "free_y") +  theme_bw() +   ylab("Predicted drop in visits") +   xlab("Week")ggsave(all_preddrop_plot, device = "pdf", filename = "all_preddrop.pdf", height = 2, width = 3, units = "in")all_preddrop_plot```## Methods### DataI figure Caitlin has most of this - as far as I've modeled things we also dropped folks from races that were not White, Black or African American, Hispanic, or Asian as they had around 4 visits per week. This would make creating tables easier. If you all would prefer leave all those folks in, I'm happy to rerun things.Then add something likeAfter exclusions, the data were grouped into either categories by race (White, Black or African American, Hispanic, and Asian) or age group (40-49, 50-59, 60-69, 70-79, and 80+). We then create weekly counts of screening mammograms beginning on Sunday, December 30th, and ending on Friday, December 31st, 2022.#### PredictorsWe use an indicator variable to describe whether or not the COVID-19 pandemic can affect screening mammogram counts. This indicator variable is set to 1 for all weeks starting on Sunday, March 1st, 2020, until August 30th, 2020. Further, we use information from the departments of Surgery and Radiology at WashU that state that, beginning March 23rd through at least May 15th, all mammograms for asymptomatic individuals were ceased as a precaution. As a result, we set the pandemic effect to full for all time points before May 15th, 2020 - which means that the first week it's allowed to vary again is the week of May 10th as the last two days are out of this time period (in which we see 6 visits). During the period where all screenings were stopped, we observe 0 visits.In order to account for the effect that holidays have on screening counts for a week, we create an indicator variable that describes if any of New Year's Day, Memorial Day, Independence Day, Labor Day, Thanksgiving Day, or Christmas Day fall into the week.To account for seasonal effects, we take a spectral decomposition approach. We allow the model to fit the two parameters that describe the ebb and flow of the seasonality trend across all groups simultaneously - one for the period, or timing of the ebb and flow, and another for the magnitude. This approach is more parsimonious than an approach that attempts to decribe patterns monthly or seasonally with a single parameter for each, and also doesn't have the same issues related to number of patient days (which is totally unknown).### ModelTo account for trends and non-independence over time, we fit a state-space model to describe screening mammogram counts in the groups of interest while incorporating effects from each of the predictors. For simplicity, we leave prior parameterization and the state portion, or the portion of the model governing the evolution of the parameters over time, to supplementary material section XX.#### The observation level modelWe fit the negative binomial regression model$$  \log(E(y_{it})) = \mu_{it} + H_{it} \times \beta_H, \text{ where}$$  $$  \boldsymbol{\mu}_t = \pi_t X_t'\boldsymbol{\beta}_t + (1 - \pi_t) X_t' \boldsymbol{\omega}_t$$and - $t$ is the time index- $i$ is the index for a group observation within a given time index $t$  - $y_{it}$ is our response for group $i$ at time $t$  - $X_t'\boldsymbol{\beta}_t$ is mean effect, including group and seasonal terms, with no pandemic at time $t$- $X_t'\boldsymbol{\omega}_t$ is mean effect, including group and seasonal terms, full pandemic at time $t$  - $\pi_{it} \in (0,1)$ is the "phase in" or "phase out" parameter that allows for the effect of the COVID pandemic to either take effect or phase out- colloquially, we say that, when $\pi_{it}=0$, we're in the pandemic. When $\pi_{it}=1$, we're not. This language isn't exact, as we're really taking a "model averaging" approach to describe what's happening, but it's close enough and doesn't impede discussing our output of interest, which is the number of missed visits- $H_{it}$ is a holiday indicator covariate for group $i$ at time $t$- $\beta_{H}$ is the effect of holidays on pandemic screeningsFor details on how the model handles $\pi_{it}$, see supplementary material XX. The above approach gives the ability to take an interrupted time series view on modeling the effect of COVID-19. #### Posterior inferencesAs we took a Bayesian approach, our interval estimates are most efficiently derived from the MCMC samples of the parameters of interest. In this work, we derive all 95% confidence intervals from the 2.5th and 97.5th percentiles of MCMC draws. We also use this same strategy to derive confidence intervals for inferences combining information from several parameters of interest.## Methodolgy concernsIn the following subsections, I will explain the methodology used in lay person's terms. I will expand or contract these sections depending on the target journal. If there are any questions about the below, I'm happy to chat about them.### Drop in visitsIn order to calculate the predicted drop in visits, we use the posterior predictive distribution and MCMC sampling to find a distribution of possible "missed visits", corresponding to a weighted average of the predictions from the two models- the no pandemic process of $\log(E(y'_{it})) = \mu'_{it} + H_{it} \times \beta_H$, where  - $\mu'_{it} = X_t'\boldsymbol{\beta}_t$, and - the observed process $\log(E(y_{it})) = \mu_{it} + H_{it} \times \beta_H$If $z_{it}'$ is the number of predicted visits in the no pandemic process and $z_{it}$ is the predicted visits in the observed model, the predicted number of missed visits weighted by the pandemic effect is then  $$\kappa_{it} = (1 - \pi_{it}) \times (z_{it}' - z_{it})$$which allows us to interpret what was happening in the context of missed visits while the pandemic was affecting the process.### Phase in and phase outIn order to allow the effect of the pandemic "phase in" and "phase out", we specify a model, per group defined by race/age group, that uses- $1-\boldsymbol{\Phi} (\alpha_g + t \times \gamma_g)=\pi_{tg}$ during the "phase in" period, or any time between March 1st 2020 and March 23rd 2020, and- $\boldsymbol{\Phi} (\eta_g + t \times \delta_g)=\pi_{tg}$ during the "phase out" period, or any time between May 15th 2020 and August 1st 2020, where$g$ is the group index for race or age group.### State-space parameterizationState-space models are a wonderful approach to time series problems, and particularly convenient to implement as a Bayesian (https://doi.org/10.1007/978-3-030-76124-0). In each of our mean processes for our groupings, which are- $\boldsymbol{\beta}_t$, or the mean effect for the age/race groups + a seasonal term out of pandemic, or- $\boldsymbol{\omega}_p$, the same during the pandemic  - subscript $p$ because we only fit this process when the pandemic may be affecting screeningsyou notice a $t$ or $p$ subscript. That's because these parameters are allowed to vary over time. Although we do not let the seasonal term vary over time, we ignore this technicality to keep confusion about what parameter describes what to a minimum. We specify the state portion of the model as                                          $$\boldsymbol{\beta}_t \sim N(\boldsymbol{\beta}_{t-1}, \Sigma_\beta) \text { and}$$$$\boldsymbol{\omega}_p \sim N(\boldsymbol{\omega}_{p-1}, \Sigma_\omega).$$                                           This allows us to account for the fact that these processes aren't time invariant - in other words, there's a "stickyness" to the process as we watch it evolve over time. This is also pretty easy to digest as a Bayesian process - we set priors for these mean effects as such$$\boldsymbol{\beta}_1 \sim N(\boldsymbol{3}, 3 \times \boldsymbol{I}), \text{ and}$$for $p=1$ at time $t'$,    $$\boldsymbol{\omega}_1 \sim N(\boldsymbol{-1} \cdot \boldsymbol{\beta}_{t'-1}, \Sigma_\omega).$$The covariance matrices $\Sigma_\beta$ and $\Sigma_\omega$ are given relatively non-informative priors that *slightly* encourage regularization.Because of this approach and our assumption that the process returns to something that resembles "normalcy" after some number of weeks, our prediction of missed visits problem represents a difference between a random walk that starts and ends in a particular spot, also known as a Brownian bridge, and the observed process.
