# Supplementary material for: Impact of COVID-19 pandemic on breast cancer screening in a large midwestern United States academic medical center
Source: PLoS One. 2024 May 20;19(5):e0303280. doi: 10.1371/journal.pone.0303280 (PMC11104587; doi:10.1371/journal.pone.0303280)
Supplement: S5 File — It takes them and wrangles them into the appropriate output for tables and figures. (RTF) [file pone.0303280.s005.rtf]

rm(list = ls())library(rstan)library(tidyverse)load("./mammo_041323.RData")holidays <- read_csv("./holidays.csv") %>%  mutate(dateh = as.Date(dateh, format = "%m/%d/%y"),         week_of = floor_date(dateh, unit = "week")) %>%  select(week_of, holiday) %>%  unique()screen_demo_final <- screen_demo_final %>%  mutate(week_of = floor_date(procedure_date, unit = "week"))count_data_prep <- function(data, holidaydf = holidays, groupname,                             pstart = "3/1/2020",                            pend = "8/1/2020") {  all_weeks <- expand_grid(groupvar = data %>%                             select(matches(groupname, ignore.case = FALSE)) %>%                             unique() %>%                             unlist(),                            # week_of = data %>%                            #   select(week_of) %>%                           #   unique() %>%                           #   unlist()) %>%                           week_of = seq(from = as.Date("12/30/2018", format = "%m/%d/%Y"),                                         to = as.Date("1/1/2022", format = "%m/%d/%Y"),                                         by = 7)) %>%    mutate(week_of = as.Date(week_of, origin = "1970-01-01")) %>%    # right_join(tibble(week_of = seq(from = as.Date("12/30/2018", format = "%m/%d/%Y"),    #                                 to = as.Date("1/1/2022", format = "%m/%d/%Y"),    #                                 by = 7))) %>%    left_join(holidaydf) %>%    mutate(holiday = case_when(      is.na(holiday) ~ 0,      TRUE ~ holiday    )) %>%    arrange(week_of)    grouped_data <- data %>%    select(week_of, groupvar = matches(groupname, ignore.case = FALSE)) %>%    group_by(week_of, groupvar) %>%    # group_by(week_of, across(matches(groupname, ignore.case = FALSE))) %>%    count() %>%    ungroup() %>%    right_join(all_weeks) %>%    mutate(n = case_when(      is.na(n) ~ 0,      TRUE ~ n    ),    pandemic = case_when(      week_of >= as.Date(pstart, format = "%m/%d/%Y") & week_of <= as.Date(pend, format = "%m/%d/%Y") ~ 1,      TRUE ~ 0    )) %>%    arrange(week_of, groupvar)        grouped_data <- grouped_data %>%    # group_by(across(matches(groupname, ignore.case = FALSE))) %>%    group_by(groupvar) %>%    mutate(tindex = row_number()) %>%    ungroup() %>%    group_by(week_of) %>%    mutate(rindex = row_number()) %>%    ungroup()    return(grouped_data)}race_data <- count_data_prep(data = screen_demo_final %>%                               # filter(!is.na(race_col)),                               filter(grepl("White|Black or African American|Hispanic|Asian",                                            # filter(grepl("White|Black or African American",                                            x = race_col),                                      !is.na(age_cat)),                             groupname = "race_col")### instead of running the above, read in "race_data.csv"write_csv(race_data, "race_data.csv")race_data_stan <- list(mammo_counts = race_data %>%                         select(n) %>% unlist(),                       tmax = max(race_data$tindex),                       gmax = max(race_data$rindex),                       kmax = max(race_data$rindex) + 1,                       pmax = race_data %>%                         filter(pandemic == 1) %>%                         select(tindex) %>% unique() %>% unlist() %>% length(),                       pstart = race_data %>%                         filter(pandemic == 1) %>%                         select(tindex) %>% unique() %>% unlist() %>% min(),                       pend = race_data %>%                         filter(pandemic == 1) %>%                         select(tindex) %>% unique() %>% unlist() %>% max(),                       X = cbind(race_data %>%                                   select(rindex) %>%                                   mutate(ones = 1,                                          rn = row_number()) %>%                                   tidyr::pivot_wider(id_cols = rn, names_from = rindex, values_from = ones, values_fill = 0) %>%                                   select(-rn) %>%                                   as.matrix(),                                 race_data %>%                                   select(holiday) %>%                                   as.matrix()),                       tindex = race_data %>% select(tindex) %>% unlist(),                       gindex = race_data %>% select(rindex) %>% unlist(),                       pno = race_data %>%                         transmute(pno = 1 - pandemic) %>%                         unlist(),                       pmaybepre = race_data %>%                         inner_join(race_data %>%                                      select(week_of, n) %>%                                      group_by(week_of) %>%                                      summarize(ntot = sum(n)) %>%                                      ungroup()) %>%                         transmute(                           pmaybe = case_when(                             pandemic == 1 & ntot > 0 ~ 1 & week_of < as.Date("4/15/2020", format = "%m/%d/%Y"),                             TRUE ~ 0                           )) %>%                         unlist(),                       pmaybepost = race_data %>%                         inner_join(race_data %>%                                      select(week_of, n) %>%                                      group_by(week_of) %>%                                      summarize(ntot = sum(n)) %>%                                      ungroup()) %>%                         transmute(pmaybe = case_when(                           pandemic == 1 & ntot > 0 ~ 1 & week_of >= as.Date("4/15/2020", format = "%m/%d/%Y"),                           TRUE ~ 0                         )) %>%                         unlist(),                       # pms = race_data %>%                       #   mutate(pmaybe = case_when(                       #     pandemic == 1 & n > 0 ~ 1 & week_of < as.Date("4/15/2020", format = "%m/%d/%Y"),                       #     TRUE ~ 0                       #   )) %>%                       #   summarize(get = sum(pmaybe * tindex) / sum(pmaybe)) %>%                       #   unlist(),                       pms = 64,                       pme = race_data %>%                         mutate(pmaybe = case_when(                           pandemic == 1 & n > 0 ~ 1 & week_of >= as.Date("4/15/2020", format = "%m/%d/%Y"),                           TRUE ~ 0                         )) %>%                         summarize(get = sum(pmaybe * tindex) / sum(pmaybe)) %>%                         unlist(),                       pdef = race_data %>%                         inner_join(race_data %>%                                      group_by(week_of, tindex) %>%                                      summarize(n = sum(n)) %>%                                      ungroup() %>%                                      mutate(pdef = case_when(                                        n == 0 ~ 1,                                        TRUE ~ 0                                      )) %>%                                       select(week_of, pdef)) %>%                         select(pdef) %>%                         unlist(),                       pnomult = c(40, 0.001),                       pmaybemult = c(10, 1),                       pdefmult = c(0.001, 40))screen_demo_final %>%  filter(!is.na(age_cat)) %>%  # filter(grepl("White|Black or African American|Hispanic|Asian",  #              # filter(grepl("White|Black or African American",  #              x = race_col)) %>%           nrow()screen_demo_final %>%  summarize(max(PROCEDURE_DATE))agec_data <- count_data_prep(data = screen_demo_final %>%                               # filter(!is.na(race_col)),                               filter(grepl("White|Black or African American|Hispanic|Asian",                                            # filter(grepl("White|Black or African American",                                            x = race_col),                                      !is.na(age_cat)),                             groupname = "age_cat")write_csv(agec_data, "age_data.csv")### instead of running the above, read in "age_data.csv"agec_data_stan <- list(mammo_counts = agec_data %>%                         select(n) %>% unlist(),                       tmax = max(agec_data$tindex),                       gmax = max(agec_data$rindex),                       kmax = max(agec_data$rindex) + 1,                       pmax = agec_data %>%                         filter(pandemic == 1) %>%                         select(tindex) %>% unique() %>% unlist() %>% length(),                       pstart = agec_data %>%                         filter(pandemic == 1) %>%                         select(tindex) %>% unique() %>% unlist() %>% min(),                       pend = agec_data %>%                         filter(pandemic == 1) %>%                         select(tindex) %>% unique() %>% unlist() %>% max(),                       X = cbind(agec_data %>%                                   select(rindex) %>%                                   mutate(ones = 1,                                          rn = row_number()) %>%                                   tidyr::pivot_wider(id_cols = rn, names_from = rindex, values_from = ones, values_fill = 0) %>%                                   select(-rn) %>%                                   as.matrix(),                                 agec_data %>%                                   select(holiday) %>%                                   as.matrix()),                       tindex = agec_data %>% select(tindex) %>% unlist(),                       gindex = agec_data %>% select(rindex) %>% unlist(),                       pno = agec_data %>%                         transmute(pno = 1 - pandemic) %>%                         unlist(),                       pmaybepre = agec_data %>%                         inner_join(agec_data %>%                                      select(week_of, n) %>%                                      group_by(week_of) %>%                                      summarize(ntot = sum(n)) %>%                                      ungroup()) %>%                         transmute(                           pmaybe = case_when(                             pandemic == 1 & ntot > 0 ~ 1 & week_of < as.Date("4/15/2020", format = "%m/%d/%Y"),                             TRUE ~ 0                           )) %>%                         unlist(),                       pmaybepost = agec_data %>%                         inner_join(agec_data %>%                                      select(week_of, n) %>%                                      group_by(week_of) %>%                                      summarize(ntot = sum(n)) %>%                                      ungroup()) %>%                         transmute(pmaybe = case_when(                           pandemic == 1 & ntot > 0 ~ 1 & week_of >= as.Date("4/15/2020", format = "%m/%d/%Y"),                           TRUE ~ 0                         )) %>%                         unlist(),                       # pms = agec_data %>%                       #   mutate(pmaybe = case_when(                       #     pandemic == 1 & n > 0 ~ 1 & week_of < as.Date("4/15/2020", format = "%m/%d/%Y"),                       #     TRUE ~ 0                       #   )) %>%                       #   summarize(get = sum(pmaybe * tindex) / sum(pmaybe)) %>%                       #   unlist(),                       pms = 64,                       pme = agec_data %>%                         mutate(pmaybe = case_when(                           pandemic == 1 & n > 0 ~ 1 & week_of >= as.Date("4/15/2020", format = "%m/%d/%Y"),                           TRUE ~ 0                         )) %>%                         summarize(get = sum(pmaybe * tindex) / sum(pmaybe)) %>%                         unlist(),                       pdef = agec_data %>%                         inner_join(agec_data %>%                                      group_by(week_of, tindex) %>%                                      summarize(n = sum(n)) %>%                                      ungroup() %>%                                      mutate(pdef = case_when(                                        n == 0 ~ 1,                                        TRUE ~ 0                                      )) %>%                                       select(week_of, pdef)) %>%                         select(pdef) %>%                         unlist(),                       pnomult = c(40, 0.001),                       pmaybemult = c(10, 1),                       pdefmult = c(0.001, 40))ever_data <- count_data_prep(data = screen_demo_final %>%                               # filter(!is.na(race_col)),                               filter(grepl("White|Black or African American|Hispanic|Asian",                                            # filter(grepl("White|Black or African American",                                            x = race_col),                                      !is.na(age_cat)) %>%                               mutate(everybody = 1),                             groupname = "everybody")ever_data_stan <- list(mammo_counts = ever_data %>%                         select(n) %>% unlist(),                       tmax = max(ever_data$tindex),                       gmax = max(ever_data$rindex),                       kmax = max(ever_data$rindex) + 1,                       pmax = ever_data %>%                         filter(pandemic == 1) %>%                         select(tindex) %>% unique() %>% unlist() %>% length(),                       pstart = ever_data %>%                         filter(pandemic == 1) %>%                         select(tindex) %>% unique() %>% unlist() %>% min(),                       pend = ever_data %>%                         filter(pandemic == 1) %>%                         select(tindex) %>% unique() %>% unlist() %>% max(),                       X = cbind(ever_data %>%                                   select(rindex) %>%                                   mutate(ones = 1,                                          rn = row_number()) %>%                                   tidyr::pivot_wider(id_cols = rn, names_from = rindex, values_from = ones, values_fill = 0) %>%                                   select(-rn) %>%                                   as.matrix(),                                 ever_data %>%                                   select(holiday) %>%                                   as.matrix()),                       tindex = ever_data %>% select(tindex) %>% unlist(),                       gindex = ever_data %>% select(rindex) %>% unlist(),                       pno = ever_data %>%                         transmute(pno = 1 - pandemic) %>%                         unlist(),                       pmaybepre = ever_data %>%                         inner_join(ever_data %>%                                      select(week_of, n) %>%                                      group_by(week_of) %>%                                      summarize(ntot = sum(n)) %>%                                      ungroup()) %>%                         transmute(                           pmaybe = case_when(                             pandemic == 1 & ntot > 0 ~ 1 & week_of < as.Date("4/15/2020", format = "%m/%d/%Y"),                             TRUE ~ 0                           )) %>%                         unlist(),                       pmaybepost = ever_data %>%                         inner_join(ever_data %>%                                      select(week_of, n) %>%                                      group_by(week_of) %>%                                      summarize(ntot = sum(n)) %>%                                      ungroup()) %>%                         transmute(pmaybe = case_when(                           pandemic == 1 & ntot > 0 ~ 1 & week_of >= as.Date("4/15/2020", format = "%m/%d/%Y"),                           TRUE ~ 0                         )) %>%                         unlist(),                       # pms = ever_data %>%                       #   mutate(pmaybe = case_when(                       #     pandemic == 1 & n > 0 ~ 1 & week_of < as.Date("4/15/2020", format = "%m/%d/%Y"),                       #     TRUE ~ 0                       #   )) %>%                       #   summarize(get = sum(pmaybe * tindex) / sum(pmaybe)) %>%                       #   unlist(),                       pms = 64,                       pme = ever_data %>%                         mutate(pmaybe = case_when(                           pandemic == 1 & n > 0 ~ 1 & week_of >= as.Date("4/15/2020", format = "%m/%d/%Y"),                           TRUE ~ 0                         )) %>%                         summarize(get = sum(pmaybe * tindex) / sum(pmaybe)) %>%                         unlist(),                       pdef = ever_data %>%                         inner_join(ever_data %>%                                      group_by(week_of, tindex) %>%                                      summarize(n = sum(n)) %>%                                      ungroup() %>%                                      mutate(pdef = case_when(                                        n == 0 ~ 1,                                        TRUE ~ 0                                      )) %>%                                       select(week_of, pdef)) %>%                         select(pdef) %>%                         unlist(),                       pnomult = c(40, 0.001),                       pmaybemult = c(10, 1),                       pdefmult = c(0.001, 40))save.image(file = "race_age_data.RData")stan_count <- "data {  int<lower=0> tmax;  int<lower=0> gmax;  int<lower=gmax> kmax;  int<lower=0> pmax;  int<lower=0> pstart;  int<lower=0> pend;  int<lower=0> tindex[tmax * gmax];  int<lower=0> gindex[tmax * gmax];  int<lower=0> mammo_counts[tmax * gmax];  matrix[tmax * gmax, kmax] X;  real pno[tmax * gmax];  real pmaybepre[tmax * gmax];  real pmaybepost[tmax * gmax];  real<lower=0> pms;  real<lower=0> pme;  real pdef[tmax * gmax];}transformed data {  matrix[tmax * gmax, kmax + 1] X_full;  matrix[tmax * gmax, kmax] X_drop; // no holiday effect    X_full = append_col(X, rep_vector(1.0, tmax * gmax));  // no holiday effect in pandemic  X_drop = append_col(block(X, 1, 1, gmax * tmax, gmax), rep_vector(1.0, tmax * gmax));}parameters {  // mean innovation  vector[gmax] omega[tmax-1];  vector[gmax] omegadrop[pmax-1];    // mean prior  vector[gmax] mu0;    // group evo covs  cholesky_factor_corr[gmax] L_Omega_mu;  vector<lower=0>[gmax] L_sigma_mu;  real<lower=0> eta; // for chol lkj  vector<lower=0>[gmax] drop_var; // drop variances    // spectral parts  real spectral_mu;  real<lower=0,upper=1> spectral_shift;    // holiday  real holiday;    // descend  vector[gmax] descend_shift0;  vector[gmax] descend_scale0;    // ascend  vector[gmax] ascend_shift0;  vector[gmax] ascend_scale0;}transformed parameters {  // means  vector[gmax] mu[tmax];    // mudrop center  vector[gmax] mudrop[pmax];    // evos  matrix[gmax,gmax] L_Sigma_mu;    // spectral  vector[tmax] spectral_t;    // betas  vector[kmax+1] betas[tmax]; // add spectral  vector[kmax] betasdrop[tmax]; // add spectral    // switch, priors informed by ascend/descend + input data  vector<lower=0,upper=1>[tmax * gmax] alpha;  vector<lower=0,upper=1>[tmax * gmax] balpha;    // betas for that prob par  real<lower=0,upper=1> alpha_mix[pmax*gmax];    // mean process  vector[tmax * gmax] mean_process;  vector[tmax * gmax] drop_process;    // get those evo vars ready  L_Sigma_mu = diag_pre_multiply(L_sigma_mu, L_Omega_mu);    // centers at - pi / 7, was close to the mean in the prelim analyses  for (t in 1:(tmax)) {    spectral_t[t] = cos(2.0 * pi() * ((1.0 / (365.25 / 7.0)) * t +                     (pi() * spectral_shift - (4.0 * pi() / 7.0))));  }    for (i in 1:(tmax * gmax)) {    alpha[i] = pno[i] +       pmaybepre[i] * (1 - Phi_approx(descend_scale0[gindex[i]] * (tindex[i] - pms) + descend_shift0[gindex[i]])) +       pmaybepost[i] * Phi_approx(ascend_scale0[gindex[i]] * (tindex[i] - pme) + ascend_shift0[gindex[i]]);    balpha[i] = pdef[i] +       pmaybepre[i] * Phi_approx(descend_scale0[gindex[i]] * (tindex[i] - pms) + descend_shift0[gindex[i]]) +       pmaybepost[i] * (1 - Phi_approx(ascend_scale0[gindex[i]] * (tindex[i] - pme) + ascend_shift0[gindex[i]]));    if (tindex[i] >= pstart && tindex[i] <= pend) {      alpha_mix[i-(pstart-1)*gmax] = alpha[i] / (balpha[i] + alpha[i]);    }  }    // centralized mean shifts  // // unaffected process  mu[1] = mu0;  for (t in 2:tmax) {    mu[t] = mu[t-1] + L_Sigma_mu' * omega[t-1];  }    // initialize at opposite of mu  mudrop[1] = -1.0 * sqrt(drop_var) .* mu[pstart-1];  for (p in 2:pmax) {    mudrop[p] = mudrop[p-1] + sqrt(drop_var) .* omegadrop[p-1];  }    // betas  for (t in 1:tmax) {    for (g in 1:gmax) {      betas[t,g] = mu[t,g];      if (t < pstart || t > pend) {        betasdrop[t,g] = 0.0;      } else {        betasdrop[t,g] = mudrop[t-pstart+1,g];      }    }    betas[t,kmax] = holiday;    betas[t,kmax + 1] = spectral_t[t] * spectral_mu;    if (t < pstart || t > pend) {      betasdrop[t,kmax] = 0.0;    } else {      // use opposite, not worth the extra effort      betasdrop[t,kmax] = -1.0 * spectral_t[t] * spectral_mu;    }  }    for (i in 1:(tmax * gmax)) {    mean_process[i] = row(X_full, i) * betas[tindex[i]];    drop_process[i] = row(X_drop, i) * betasdrop[tindex[i]];  }}model {  // corr matrices  eta ~ inv_gamma(2, 1); // incentivize regularization  L_Omega_mu ~ lkj_corr_cholesky(eta);  L_sigma_mu ~ inv_gamma(1, 1);  drop_var ~ inv_gamma(1, 1);    // spectral shift  spectral_shift ~ beta(3, 3);    // mean components, initialize  // spectral mu  spectral_mu ~ normal(0, 3);  // reg mu  mu0 ~ normal(2, 3);    // drop process    // holiday  holiday ~ normal(0, 3);    // centralized innovation  for (t in 1:(tmax-1)) {    omega[t] ~ normal(0, 1);  }  // innovation variance for drop  for (p in 1:(pmax-1)) {    omegadrop[p] ~ normal(0, 1);  }    // ascend/descend  descend_shift0 ~ normal(0, 10);   ascend_shift0 ~ normal(0, 10);   descend_scale0 ~ normal(0, 10);   ascend_scale0 ~ normal(0, 10);    // no and def probs  for (i in 1:(tmax * gmax)) {    if (tindex[i] < pstart || tindex[i] > pend) {      target += poisson_log_lpmf(mammo_counts[i] | mean_process[i]);    } else {      // alpha_mix[i-(pstart-1)*gmax] ~ beta(alpha[i], balpha[i]);      target +=  poisson_log_lpmf(mammo_counts[i] |                    alpha_mix[i-(pstart-1)*gmax] * mean_process[i] +                      (1 - alpha_mix[i-(pstart-1)*gmax]) * drop_process[i]);    }  }}generated quantities {  int<lower=0> np_counts[tmax * gmax];  int<lower=0> pred_counts[tmax * gmax];  int pred_diff[tmax * gmax];  int obs_diff[tmax * gmax];    for (i in 1:(tmax * gmax)) {    if (mean_process[i] < 20.0) {      np_counts[i] = poisson_log_rng(mean_process[i]);      } else {      np_counts[i] = poisson_log_rng(20.0);    }    if (tindex[i] < pstart || tindex[i] > pend) {      pred_counts[i] = np_counts[i];    } else {      if ((alpha_mix[i-(pstart-1)*gmax] * mean_process[i] +          (1 - alpha_mix[i-(pstart-1)*gmax]) * drop_process[i]) < 20.0) {        pred_counts[i] = poisson_log_rng(alpha_mix[i-(pstart-1)*gmax] * mean_process[i] +           (1 - alpha_mix[i-(pstart-1)*gmax]) * drop_process[i]);      } else {        pred_counts[i] = poisson_log_rng(20.0);      }    }          pred_diff[i] = np_counts[i] - pred_counts[i];    obs_diff[i] = np_counts[i] - mammo_counts[i];  }}"options(mc.cores = parallel::detectCores())fit_race <- stan(model_code = stan_count,                  model_name = "pois_race_mammo",                  data = race_data_stan,                  chains = 4,                  iter = 1500,                  warmup = 1000,                 seed = 1997)#,                  # control = list(adapt_delta = 0.95,                  #                stepsize = 0.5))#,                                 #max_treedepth = 15))save(fit_race, file = "new_race_res.RData")fit_agec <- stan(model_code = stan_count,                  model_name = "pois_agec_mammo",                  data = agec_data_stan,                  chains = 4,                  iter = 1500,                  warmup = 1000,                 seed = 1998,                 control = list(adapt_delta = 0.85))save(fit_agec, file = "new_agec_res.RData")### everybody nowfit_ever <- stan(model_code = stan_count,                  model_name = "pois_ever_mammo",                  data = ever_data_stan,                  chains = 4,                  iter = 1500,                  warmup = 1000,                 seed = 1999,                 control = list(adapt_delta = 0.99,                                stepsize = 0.5))save(fit_ever, file = "new_ever_res.RData")
